# Enhanced phenol removal from wastewater via sulfuric acid activated eggshell derived carbon

**DOI:** 10.1038/s41598-025-04615-y

**Published:** 2025-06-20

**Authors:** Heba A. El-Gawad, Mostafa H. Hussein, Hamdy A. Zahran, Ghada Kadry

**Affiliations:** 1 Department of Engineering Mathematics and Physics, Higher Institute of Engineering, El-Shorouk Academy, Cairo, Egypt; 2 Chemical Engineering Department, Higher Institute of Engineering, El- Shorouk Academy, Cairo, Egypt; 3 Fats and Oils Department, Food Industries and Nutrition Research Institute, National Research Centre, Dokki, Cairo Egypt

**Keywords:** Phenol adsorption, Eggshell-derived activated carbon, Response surface methodology (RSM), Kinetic adsorption studies, Isothermal adsorption studies, Adsorption mechanisms, Activation process, Environmental sciences, Environmental chemistry, Environmental impact

## Abstract

This research explores the development of an innovative activated carbon adsorbent (ACES) derived from waste eggshells through sulfuric acid activation to effectively remove phenol from simulated wastewater. Optimization of adsorption parameters was conducted using Design-Expert 13 software and response surface methodology (RSM). Under optimal conditions (initial phenol concentration of 25.015 mg/L, adsorbent dosage of 4.913 g/L, pH of 4.693, and temperature of 25.013 °C), ACES achieved an outstanding phenol removal efficiency of 99.87%. Characterization studies revealed a high BET surface area of 1034.775 m²/g and enhanced porosity, significantly contributing to adsorption performance. Mechanistic insights showed that electrostatic attraction, π–π interactions, and hydrogen bonding drove adsorption. The Langmuir model provided the best fit for phenol adsorption on ACES (R² = 0.9845), indicating monolayer adsorption on uniform sites. Kinetic analysis revealed that the adsorption followed pseudo-second-order kinetics, with a rate constant (k) of 0.0078 g·min⁻¹·mg⁻¹ and a high correlation coefficient (R² = 0.9886), pointing to chemisorption rather than physical adsorption. Thermodynamic analysis further confirmed that the process is spontaneous and exothermic, accompanied by increased randomness at the adsorbent-adsorbate interface. ACES exhibited good reusability, retaining 80% efficiency after four regeneration cycles. The findings of this research highlight a sustainable approach to utilizing waste eggshells for phenol removal, offering potential applications in wastewater treatment.

## Introduction

Phenol, a prominent pollutant found in the wastewater of various industries, poses significant health risks^1^. It is commonly present in the effluent of processing units involved in the production of phenolic resins, epoxy resins, adhesives, and other materials^2–4^. Phenolic contaminants are categorized as priority contaminants because of their detrimental impact on organisms and aquatic life, in addition to their potential carcinogenic properties. Their stability and water solubility make them particularly challenging to decompose, leading to unpleasant odours and toxicity^2,5,6^. Consequently, wastewater containing phenolic compounds necessitates effective treatment before environmental discharge^3,7^.

Several initiatives have been undertaken to devise efficient approaches for removing phenol from wastewater. Diverse physicochemical treatment processes, including adsorption, ion exchange, reverse osmosis, precipitation, coagulation, and filtration, along with chemical techniques like photocatalytic oxidation, ozone treatment, and Fenton’s process, as well as biodegradation techniques involving fungi, algae, bacteria, and microbial fuel cells, have been explored^8^. However, these methods are often expensive, energy-demanding, non-specific, involve hazardous materials, and lack scalability and efficiency^6,9,10^. Secondary biological treatment processes struggle to handle high concentrations of phenolic wastewaters^3^.

Recently, there has been a growing emphasis on creating eco-friendly, affordable, and efficient approaches to remove phenol from wastewater. Among these methods, adsorption has become a leading technique for water purification due to its simplicity, efficiency, and cost-effectiveness^9,11–15^. Selecting a suitable adsorbent is crucial for both pilot and industrial-scale wastewater treatment. An effective adsorbent should have a high adsorption capacity, selectivity, fast adsorption and desorption kinetics, and require minimal energy for regeneration to efficiently remove contaminants^12^. Activated carbon, a well-established adsorbent, exhibits excellent phenol adsorption capacities^16–18^. However, the high cost of commercial activated carbon has prompted researchers to explore alternative, low-cost precursors^13^. Traditional activated carbon production relies on expensive and non-renewable sources like wood waste, coal, and petroleum residues^19–21^. To address this, researchers have turned to sustainable and affordable materials, including agricultural residues (rice husk, corn straw, sugarcane bagasse), natural clay, and waste materials like eggshells^13,22–26^. These materials can be activated through physical or chemical methods. Physical activation involves carbonization followed by gas activation at high temperatures, while chemical activation involves impregnation with a chemical agent and subsequent thermal treatment. Chemical activation often yields activated carbon with superior properties, including higher surface area and better-developed porosity, and typically requires less time than physical activation^27–31^. By utilizing these low-cost and sustainable precursors, researchers aim to develop cost-effective and environmentally friendly activated carbon for water purification applications^32^.

Eggshells, a readily available, eco-friendly and low-cost waste material, offer a sustainable solution for environmental remediation. Rich in calcium carbonate, eggshells can be transformed into valuable activated carbon through processes like physical or chemical (pyrolysis) activation. This transformation not only reduces waste but also produces a highly porous material capable of adsorbing various pollutants from water and wastewater.

Sulfuric acid is favoured as an activating agent in the chemical activation process due to its high acidity, effectiveness in eliminating impurities and breaking down complex organic compounds, creating a porous structure with a large surface area, which is ideal for adsorption, such as in activated carbon production^33^. It acts as a catalyst, promoting carbonization and pore formation without being consumed, improving activation process efficiency. Its low cost and availability make it an economical choice over alternatives like phosphoric acid and potassium hydroxide, especially for large-scale use. Additionally, its strong dehydrating properties aid in creating a porous structure with a high surface area during carbonization and activation processes, which a suitable for adsorption. The activation process with sulfuric acid is simple and efficient, allowing for faster breakdown of impurities and better pore development compared with agents that may require longer processing times or more specific conditions to achieve similar results.

Although sulfuric acid is corrosive, it is well-understood and manageable with proper precautions. The main environmental concern is sulphur dioxide (SO₂) emissions, which can be controlled through ventilation and neutralization. Phosphoric acid has a lower environmental impact, is more costly, and harder to dispose of, while potassium hydroxide, though free from harmful gases, is caustic and creates waste needing neutralization. Both alternatives may involve more complicated handling and additional neutralization steps^34^. Ultimately, the choice of activating agent depends on the activation process requirements, production scale, and environmental management.

Beyond their traditional culinary use, eggshells have found applications in diverse fields, including cosmetics^35^, cement production^36^, polymer and metal composites^37^, fertilizer additives, and livestock feed supplements^38^. Their porous structure, characterized by numerous pores, makes them suitable for adsorption processes to treat contaminated water and soil^39,40^. Researchers have explored the potential of eggshells and their derivatives as adsorbents for removing a wide range of pollutants, such as dyes and other organic compounds^41–45^, heavy metals^46–50^, and anions and oxyanions^51–54^.

Numerous studies have investigated the efficacy of eggshells as adsorbents for phenol removal from wastewater. For instance, Daraei et al.^55^ demonstrated the effective use of eggshell powder as an adsorbent for phenol, achieving a maximum adsorption capacity (0.45 mg/g) at optimum conditions (pH 9, 25 °C,15 mg/L initial phenol concentration, 25 °C and the contact time, 90 min). Similarly, Liliana Giraldo et al.^56^ synthesized activated carbons from chicken eggshells through physical activation methods to adsorb phenol. The process involved activating different parts of the eggshell, including shells with intact membranes (CES1), separated shells (CES), and isolated membranes (CEMemb). The resulting materials exhibited well-developed pore structures and high adsorption capacities, with surface areas ranging from 58 to 113 m²/g and pore volumes between 0.54 and 0.97 cm³/g. Under optimal conditions (500 mg adsorbent, 50 mL solution, pH 5.7, 25 °C, and 48 h), maximum adsorption capacities were 119 mg/g for CES1, 143 mg/g for CES, and 192 mg/g for CEMemb. Additionally, Chraibi et al.^57^ utilized calcined eggshell waste to remove phenol from aqueous solutions, achieving a maximum removal efficiency of 37% at a calcination temperature of 1000 °C. Kashi^58^ further explored the use of powdered eggshells with a specific surface area of 7.43 m²/g for phenol removal from drinking water, reporting a maximum adsorption capacity of 4 mg/g under optimized conditions (contact time of 80 min, initial phenol concentration of 5 mg/L, adsorbent dosage of 4 g/L, and pH of 3). These studies collectively highlight the potential of eggshells as a sustainable and effective adsorbent for wastewater treatment.

This study focuses on the synthesis and characterization of activated carbon derived from waste eggshells using sulfuric acid activation, aiming to enhance phenol removal from simulated wastewater. The research systematically investigates the effects of key operational parameters-including initial phenol concentration, adsorbent dosage, pH, and temperature-on adsorption performance, utilizing response surface methodology (RSM) for optimization. Comprehensive characterization of the prepared adsorbent is conducted using BET surface area analysis, FTIR, SEM, EDX, TGA, TEM, XRD, and Zeta potential measurements to elucidate its structural and functional properties. The adsorption mechanisms are explored through isotherm, kinetic, and thermodynamic studies, with particular attention to electrostatic interactions, π-π interactions, and hydrogen bonding. Additionally, the study evaluates the reusability of the eggshell-derived activated carbon and compares its performance to commercial alternatives. The overall goal is to provide a sustainable, efficient, and cost-effective approach for phenol removal from wastewater, highlighting the practical applicability of waste eggshells as a precursor for high-performance adsorbents.

## Materials

High-purity analytical-grade chemicals were procured to ensure experimental reliability. Sulfuric acid (H₂SO₄, 98%), sodium hydroxide (NaOH, 98%), hydrochloric acid (HCl, 30%), and phenol (C_6_H_6_O, 99%) were obtained from Pure Egypt Company. Deionized water was used throughout the study to prepare solutions. Waste eggshells, sourced locally from Cairo, Egypt, were used as the precursor material for activated carbon synthesis.

### Preparation of activated carbon

Eggshells were washed thoroughly with tap water and deionized water to eliminate impurities, then oven-dried at 105 °C for 2 h. Notably, temperatures above 105 °C could harm the calcium carbonate structure, potentially reducing phenol removal, while temperatures below 105 °C might introduce undesirable taste and odour to treated water^58^. Dried eggshells were ground into a fine powder (particle size below 250 μm) using a grinding machine and then sieved through a mechanical sieve.

The sieved material was impregnated with 70% sulfuric acid (H₂SO₄) at a 1:2 weight-to-volume ratio and left to soak overnight with continuous stirring and left overnight to activate the adsorption sites and boost the adsorption efficiency. The impregnated material was rinsed with deionized water until the pH of the filtrate reached neutrality, filtered, and dried again at 105 °C. As per Li et al. ^59^, porosity decreases with increasing temperature due to heat affecting the porous structure. Hence, carbonization was performed at ~ 650 °C, based on the TGA analysis, with a heating rate of 10 °C.min^− 1^ for 3 h in a muffle furnace (Carbolite ELF 11/14B, UK) under a nitrogen atmosphere. The resulting activated carbon was first washed with 1 M HCl to remove residual ash, followed by a thorough deionized water wash to eliminate any remaining acid traces, then dried overnight at 105 °C and stored in an airtight container for further use.

### Characterization of activated carbon

The moisture content of ACES was assessed by heating 5 gm of the material at 110 °C in a pre-weighed crucible (Initially 12.47 gm). After cooling in a desiccator, the sample and crucible were reweighed, and moisture content was computed using the formula: Moisture content = ((W_1_ + W_2_) – W_3_) / W_2_ * 100 ^59^. Here, W_1_ represents the empty crucible weight, W_2_ is the ACES weight, and W_3_ is the residue weight post-drying at 110 °C. ACES’s bulk density was assessed by measuring its volume displacement in a 100 mL graduated cylinder. This displacement, converted to mass, yielded the bulk density as: Bulk density = M / V^59^, with M being the weight (in grams) of ACES in the cylinder and V representing the volume it occupies in the cylinder.

Additionally, the Zeta potential of the adsorbent was determined using an SZ-100 nanoparticle size and Zeta potential analyzer. Crystallographic structure, chemical composition, and physical properties of the adsorbent were analysed with an X-ray diffraction (XRD) using an X’Pert High Score PANalytical XRD instrument (Malvern, UK) operating at 40 kV and 30 mA. The electronic structures of the adsorbent were examined using a Transmission Electron Microscope (TEM) - F200i TEM (20–200 kV). The identification of functional groups on the adsorbent was carried out using Fourier transform infrared (FTIR) spectroscopy ((Nicolet is10, USA) in the range of 400–4000 cm⁻¹. Thermogravimetric analysis (TGA) (TA Instruments, US) is a valuable method for assessing the thermal stability of various materials, including polymers, under a nitrogen atmosphere. Scanning Electron Microscopy (SEM) was utilized to study the surface morphology of the adsorbent using a Quanta FEG 250 SEM (Thermo Fisher Scientific, USA). Furthermore, the elemental composition analysis was conducted through Energy Dispersive X-ray (EDX) analysis using a Quanta FEG-250 SEM instrument. BET- specific surface area and pore size distribution of ACES were assessed using the Brunauer-Emmett-Teller (BET) method with an Anton Paar NOVA 800 device in Egypt, utilizing nitrogen adsorption at 77.35 K. Prior to analysis, a 0.061 g sample underwent degassing under vacuum conditions at 300 °C for 2 h, with the temperature ramping up at a rate of 20.0 °C/min.

### Adsorption experiment

A stock phenol solution of 100 ppm was prepared by dissolving 0.1 gm of phenol in 1 L of deionized water. Working solutions of 25, 50, and 75 ppm were obtained through appropriate dilutions. Batch adsorption experiments were conducted in 250 mL Erlenmeyer flasks containing 50 mL of phenol solution and the required dosage of activated carbon.

**Fig. 1:**
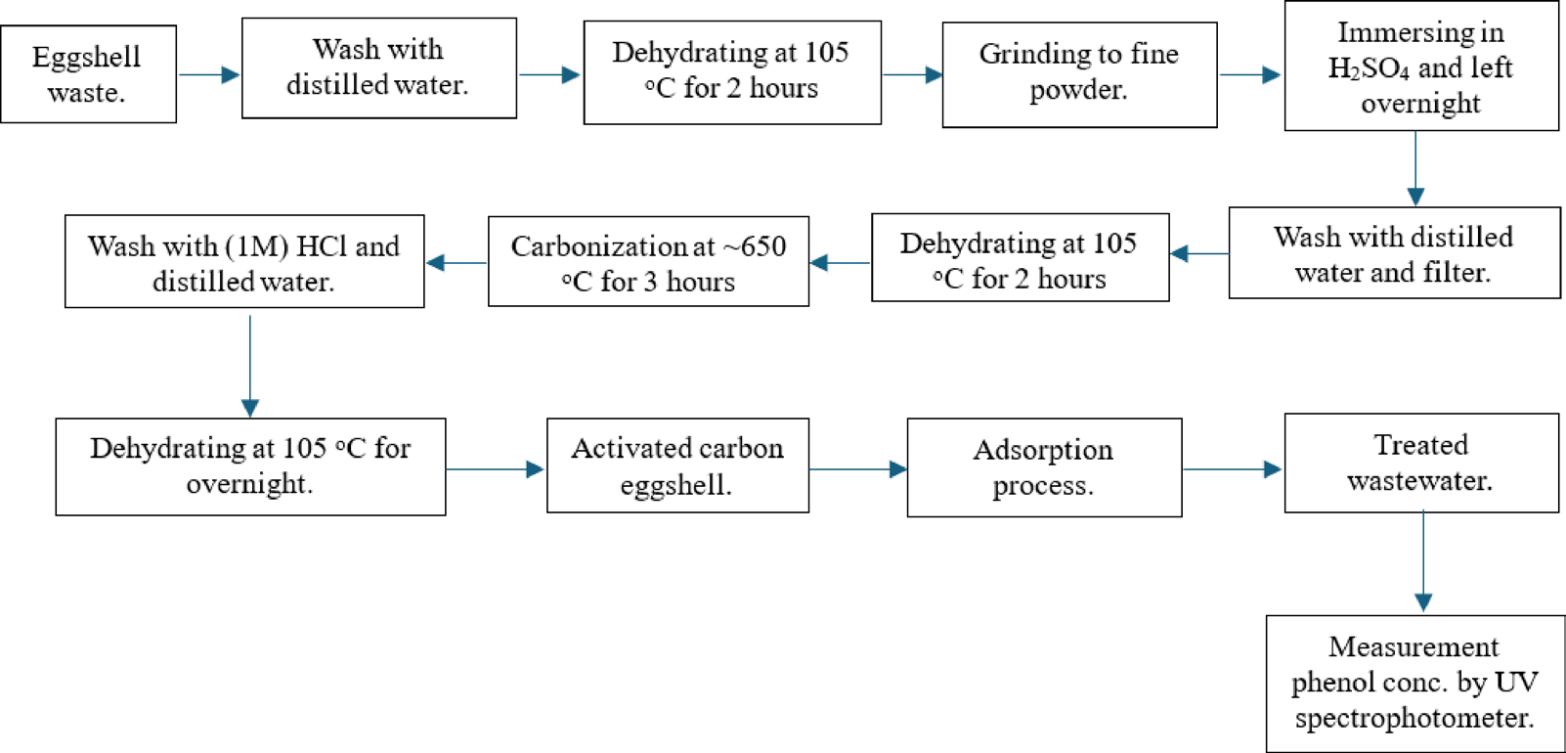
A schematic diagram illustrating the preparation process of activated carbon eggshells (ACES) and phenol adsorption from simulated wastewater into activated carbon eggshells (ACES).

The initial pH of the solutions was adjusted using 0.1 M NaOH or 0.1 M HCl solution and measured with a JENWAY pH meter (Model 3505, UK). The flasks were shaken at 300 rpm in a temperature-controlled orbital shaker (SKU: SI-G1500) for 2 h. Samples were withdrawn at specific intervals, filtered using Whatman No. 1 filter paper, and analysed for residual phenol concentration using a UV-Vis spectrophotometer (Shimadzu UV-2600, Japan) at 270 nm. A schematic diagram depicting the preparation process of activated carbon eggshells (ACES) and the subsequent adsorption of phenol from simulated wastewater onto ACES is presented in Fig. 1.

### Optimization and modelling

Design-Expert software (version 13, USA) was employed to implement a response surface methodology (RSM) along with a central composite design (CCD) for optimizing phenol removal through adsorption. Six operational factors, namely Six operational factors, namely initial phenol concentration (25–100 ppm), pH (3–9), adsorbent dosage (1–5 g/L), and temperature (25–75 °C), were scrutinized, were scrutinized. Following CCD optimization, the experiment was conducted under the predicted optimal conditions, comparing the response to predicted values. The numerical optimization process involved choosing the desired objective for each variable and response among available options. This allowed the determination of optimal conditions for maximum phenol elimination. The phenol elimination efficiency (Elimination%) and adsorption capacity (Q, mg/gm) were calculated using the following equations:

1 $$Q = [C_0-C_e] * V/ m$$

2 $$\text{Elimination }\% = [C_0-C_t]/C_0\times100$$

The relationship between the initial phenol concentration (C_₀_, mg/L), its concentration at a specific time point (C_t_, mg/L), and its equilibrium concentration (C_e_, mg/L) is captured by this equation, which also incorporates the solution volume (V, L) and ACES mass (m, gm).

## Results and discussion

The physicochemical characteristics of ACES with H_2_SO_4_ were determined, including moisture content and bulk density. To analyse the adsorbent’s structural, morphological, specific surface area, and electronic features, various techniques were employed, such as FTIR, SEM, EDX, BET Surface area, XRF, zeta potential, and TEM.

(RSM) was applied to optimize the adsorption parameters for phenol elimination throughout the entire process. By investigating the adsorption process under diverse conditions, the purpose was to define the most favourable combination of these parameters for the competence of phenol disposal.

The kinetic and isotherm models and Thermodynamic analysis were analysed using linear regression in Origin 2018 Software. To assess the adequacy of each isotherm and kinetic model quantitatively, various error metrics, including ERRSQ, RSS, ARE, and determination coefficients R² (COD) and adjusted R², were applied.

### Adsorbent characterization

#### Moisture content and bulk density

The remarkable surface area, porous structure, and natural chemical properties of acid-treated ACES enhance its phenol removal capabilities^60,61^. A preliminary analysis of ACES powder with a particle size below 250 μm revealed key physicochemical attributes, such as a moisture content of 5.3% and a bulk density of 1.07 gm/mL.

#### BET specific surface area

ACES demonstrates superior adsorption properties, exhibiting a BET-specific surface area of 1034.775 m²/g, with a pore volume of 0.82 cm³/g and an average pore diameter of 2.05 nm, indicating the presence of mesoporous structures. The BET specific surface area of ACES is approximately that of commercial activated carbons commonly documented in the literature^62^, such as Norit 1240 (around 1100 m²/g) and Filtrasorb 400 (~ 1050 m²/g). In contrast, the BET specific surface area of ACES surpasses that of several commercial activated carbons. For instance, the values for ICI Hydrodarco 3000 range between 300 and 600 m²/g, while Calgon Filtrasorb 300 and Westvaco Nuchar WL have specific surface areas of approximately 950–1050 m²/g and 1000 m²/g, respectively^63^. With a BET surface area of 1034.755 m²/g and a phenol removal efficiency of 99.87%, ACES are comparable to commercial activated carbons, offering a cost-effective and sustainable alternative. Moreover, this value substantially surpasses that of numerous biomass-derived porous carbon phenol adsorbents as reported in Table 1. The high surface area and porosity provide ample adsorption sites, enhancing the phenol removal efficiency. The increase in surface area and pore volume results from the efficient activation process using sulfuric acid, which removes volatile components and enhances porosity. These features contribute to the superior adsorption performance of ACES. The results highlight the superior performance of the activated carbon produced in this study for phenol removal from aqueous solutions compared to other adsorbents. Research studies have shown that phosphoric acid impregnation results higher surface area than potassium hydroxide because phosphoric acid molecules transform into phosphorus compounds that speed up carbonization^32^. However, sulfuric acid impregnation produces the highest surface area overall, making it the most efficient method for developing adsorbents with specific properties for adsorption purposes.

**Table 1:** Comparison of BET surface area of various activated carbons (ACs).

Adsorbent	BET specific surface area (m^2^/g)	References
ACES	1034.775	This study
Eggshell derived carbon	113	^56^
Coffee residue-based AC	520–810	^64^
Eggshell-Catalysed Biochar	621	^65^
Saccharum officinarum biomass activated carbon	415.96	^66^
Coconut shell biochar	567	^67^
Waste tire-based activated carbon (WTAC)	257	^68^
Rice husks-based AC	480	^69^
Sugarcane bagasse-base AC	709.3	^70^
Biocarbon derived from blackberry seeds	65	^71^
• Cane pith-based AC • Plum kernel-based AC • Corn cob- based AC	445 to 607 353.6 to 824.8 537.7 to 943.3	^63^
• Sugar cane bagasse-based AC • Corn cob-based AC	3 to 369 5 to 778	^72^

#### IR Spectra–Fourier transforms infrared

**Fig. 2:**
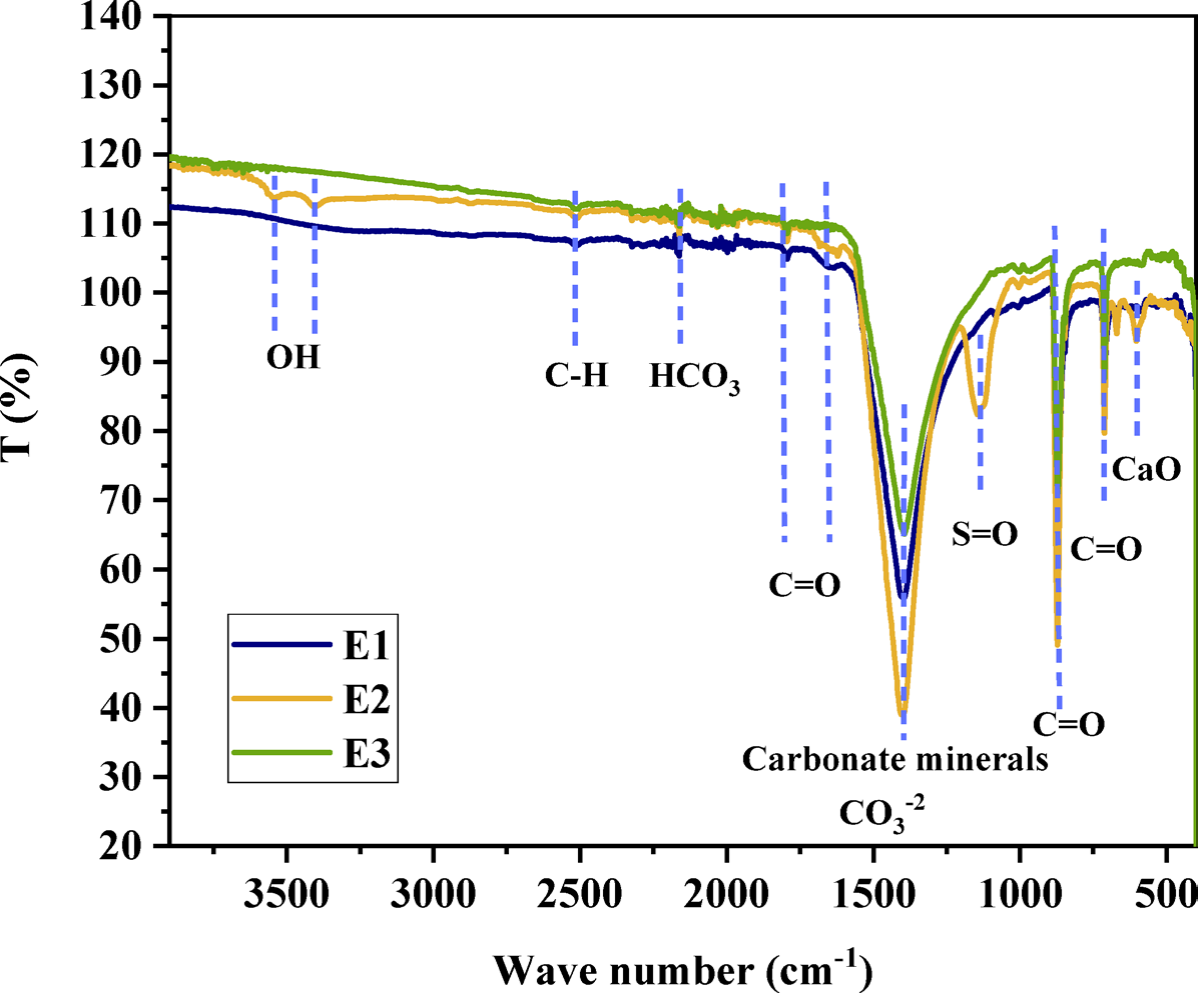
FTIR spectra of eggshell: (E1) Raw material, (E2) Pre-treated with H_2_SO_4_, and (E3) ACES at 650 °C for 3 h before adsorption.

FTIR analysis (400–4000 cm^− 1^) was conducted on raw eggshell (E1), eggshell pretreated with H_2_SO_4_ (E2) and Activated Carbon Eggshell (ACES) (E3) as exposed in Fig. 2. Key CaCO_3_ characteristics include peaks at 712, 871, 1397, and 2162 cm^− 1^. Absorption peaks in 1792 and 1639 cm^− 1^ are attributed to the stretching vibrations of the C = O bonds in carbonate ions (CO_3_ ^− 2^). The intense peak at 1397 cm^− 1^ signifies carbonate C–O bonds, while 2162 cm^− 1^ represents C-H vibrations of HCO_3_^−^. Peaks at 871 and 712 cm^− 1^ indicate bending vibration of C = O in (CO_3_ ^− 2^) molecules. C-H vibrations related to organic matter are indicated around 2510 cm^− 1 73–75^. After pretreatment with H_2_SO_4_ (E2), the spectra (Fig. 2) show minimal changes, except for a slight decrease in (CO_3_ ^− 2^) bands and C-H vibrations of organic compounds on the eggshell. New peaks at 3538 and 3412 cm^− 1^, attributed to O-H stretching (moisture), and S = O stretching from the sulfonic acid in the pretreatment appear around 1144 cm^− 1^. A new peak at approximately 603 cm^− 1^ represents CaO, a result of sulfuric acid’s chemical conversion of CaCO_3_ in eggshells^76–78^. Following activation at ~ 650 °C, the FTIR spectra of E3 reveal the removal of O-H groups. However, organic matter, carbonate minerals, and CaO groups are not eliminated by the 3-hour, ~ 650 °C activation process^79–81^.

**Fig. 3:**
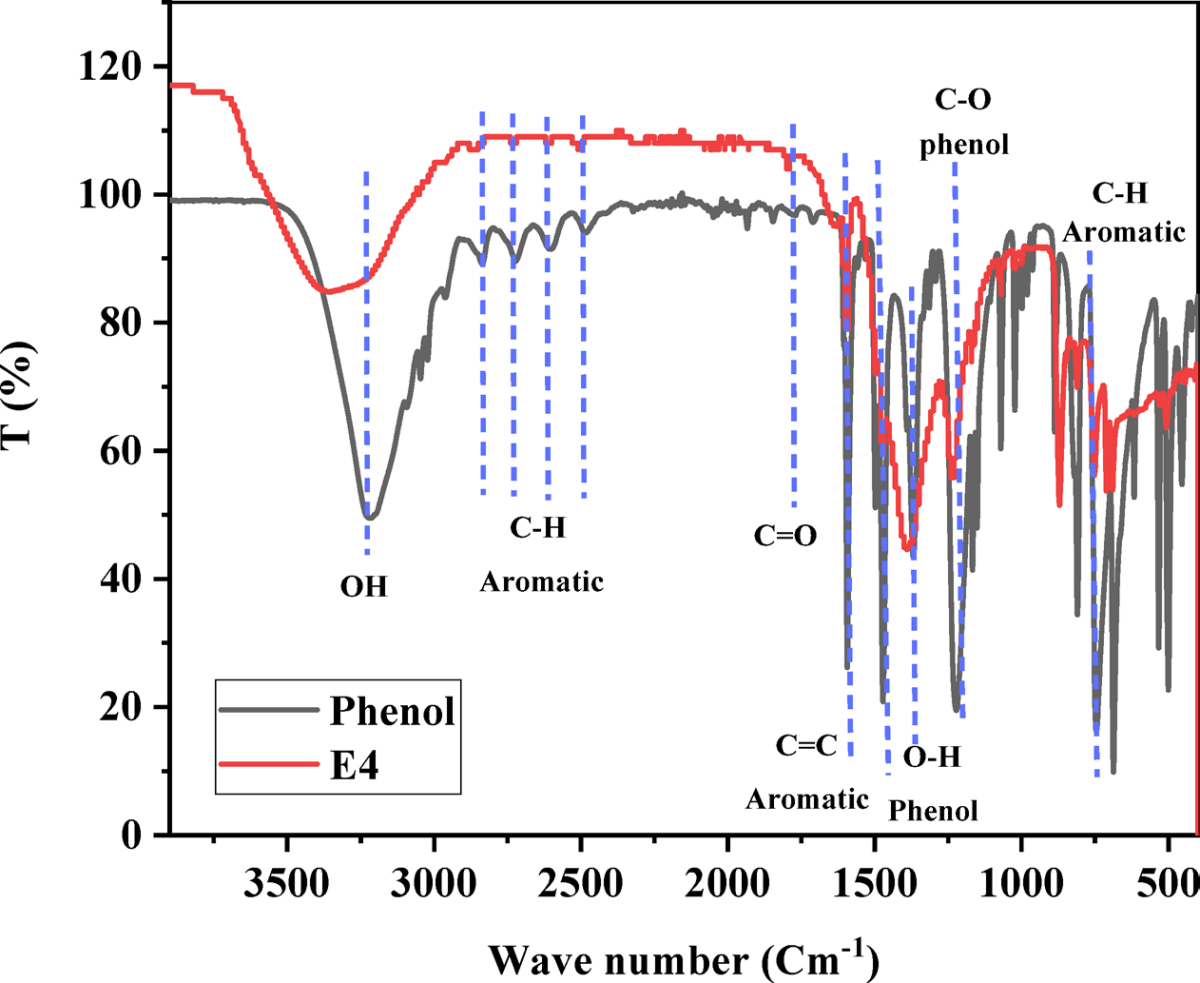
FTIR spectra of Phenol and ACES after the adsorption process (E4).

**Fig. 4:**
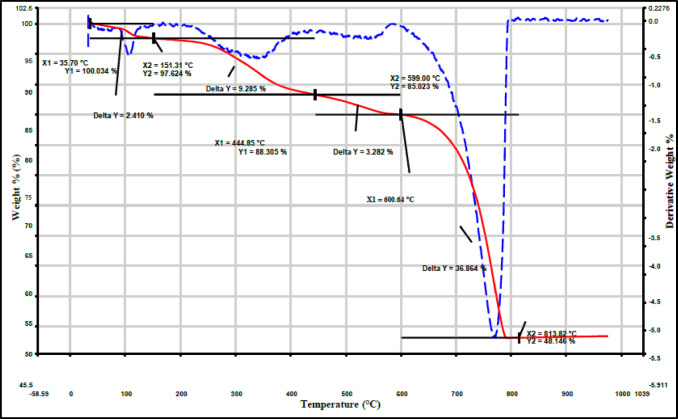
Thermogravimetric analysis (TGA) of pretreated eggshells in H_2_SO_4_ (E2).

Phenol’s structure, consisting of an aromatic ring and a hydroxyl group, is evident in its IR spectrum E4 (Fig. 3). The aromatic ring contributes to absorption bands associated with C = C stretching vibrations at 1593 and 1471 cm^− 1^. Distinctive C-H stretching vibrations of the aromatic ring appear at approximately 2715 cm^− 1^, alongside bending absorptions at 745 cm^− 1^. The hydroxyl group in phenol results in a prominent O-H stretching vibration, appearing as a broad and intense band at 3213 cm^− 1^. This broad band suggests hydrogen bonding between phenol molecules and the ACES surface. Phenol also exhibits O-H bending absorptions at 1370 cm^− 1^ (free hydroxyl groups) and a lower frequency band around 1070 cm^− 1^ (hydrogen-bonded hydroxyl groups). The fingerprint absorptions below 1600 cm^− 1^ are unique to phenol, resulting from combinations of C-O, C-C, and C-H bending vibrations, enabling phenol’s differentiation from other aromatic compounds^82–85^. The O-H stretching vibration of phenol shifts to 3361 cm^− 1^ and decreases in intensity after adsorption on the ACES, indicating the phenol OH group’s involvement in adsorption. Phenol’s aromatic C = C stretching vibrations at 1593 cm^− 1^ and 1471 cm^− 1^ decrease and shift to 1594 and 1498 cm^− 1^ as the aromatic ring engages in π-π interactions with the ACES surface. Carbonate bands from eggshell-derived activated carbon around 1390 cm^− 1^ and 871 –751 cm^− 1^ decrease in intensity, suggesting surface calcium ions interact with the phenol (OH group), forming hydrogen bonds. The S = O adsorption peaks at 1010 cm^− 1^ shift to 1168 cm^− 1^ and decrease in intensity as CaO shifts from 600 cm^− 1^ to 690 cm^− 1^ due to the interaction of the phenol (OH group) with the ACES surface. These shifts indicate the formation of surface complexes between phenol and the activated carbon surface (Fig. 4)^86–89^.

#### Thermogravimetric analysis (TGA)

In Fig. 4, TGA analysis of H_2_SO_4_ pretreated eggshells (E2) is depicted. The analysis involved heating the eggshell sample from 25 °C to 900 °C at a rate of 10 °C/min under a nitrogen atmosphere. The TGA curve reveals three distinct mass loss regions. The initial minor weight loss at 151 °C (2.4% loss) is attributed to moisture evaporation. The second loss at 444 °C (11.6% loss) corresponds to the decomposition and oxidation of organic components in the eggshell. The primary eggshell component, calcium carbonate, undergoes significant decomposition between 600 and 850 °C, releasing carbon dioxide (CO_2_) and forming calcium oxide (CaO), as depicted by chemical reaction (1)^73,90–92^. Decomposition is completed at 813 °C, indicating it as the maximum calcination temperature for pretreated eggshell. The final mass loss at 813 °C is associated with the release of residual carbonate (CaO and CO_2_) and sulphate (SO_2_) present in the pretreated eggshell (CaO/CaS, SO_2_, and CO_2_) as per chemical reactions 1 and 2,^93–95^. The total mass loss over the entire temperature range is 51.7%. The remaining mass after heating to 900 °C represents the non-volatile inorganic ash content, which is reduced by the pretreatment process through mineral dissolution.

The chemical reaction mechanism (1): thermal degradation of eggshell

$$\begin{aligned} & {\text{CaCO}}_{{\text{3}}} \left( {\text{s}} \right) \to {\text{ CaO }} + {\text{ CO}}_{{{\text{2}}({\text{g}})}} \hfill \\ & {\text{2CO}}_{{\text{2}}} \left( {\text{g}} \right) \to {\text{ 2C }} + {\text{ 2O}}_{{{\text{2}}({\text{g}})}} \hfill \\ & {\text{2CO}}_{{\text{2}}} \left( {\text{g}} \right) \to {\text{ 2CO }} + {\text{ O}}_{{{\text{2}}({\text{g}})}} \hfill \\ \end{aligned}$$

The chemical reaction mechanism (2): Thermal degradation of pretreatment eggshell with H_2_SO_4_^96^:

$$\begin{aligned} &{\text{CaCO}}_{{\text{3}}} + {\text{ H}}_{{\text{2}}} {\text{SO}}_{{\text{4}}} \to {\text{ CaSO}}_{{\text{4}}} + {\text{CO}}_{{\text{2}}} + {\text{H}}_{{\text{2}}} {\text{O}} \hfill \\ & {\text{CaSO}}_{{\text{4}}} + {\text{ CO }} \to {\text{ CaO }} + {\text{CO}}_{{\text{2}}} + {\text{ SO}}_{{\text{2}}} \hfill \\ & {\text{CaSO}}_{{\text{4}}} + {\text{ 4CO }} \to {\text{ CaS }} + {\text{ 4CO}}_{{\text{2}}} \end{aligned}$$

#### Scanning Electron microscopy (SEM)

**Fig. 5:**
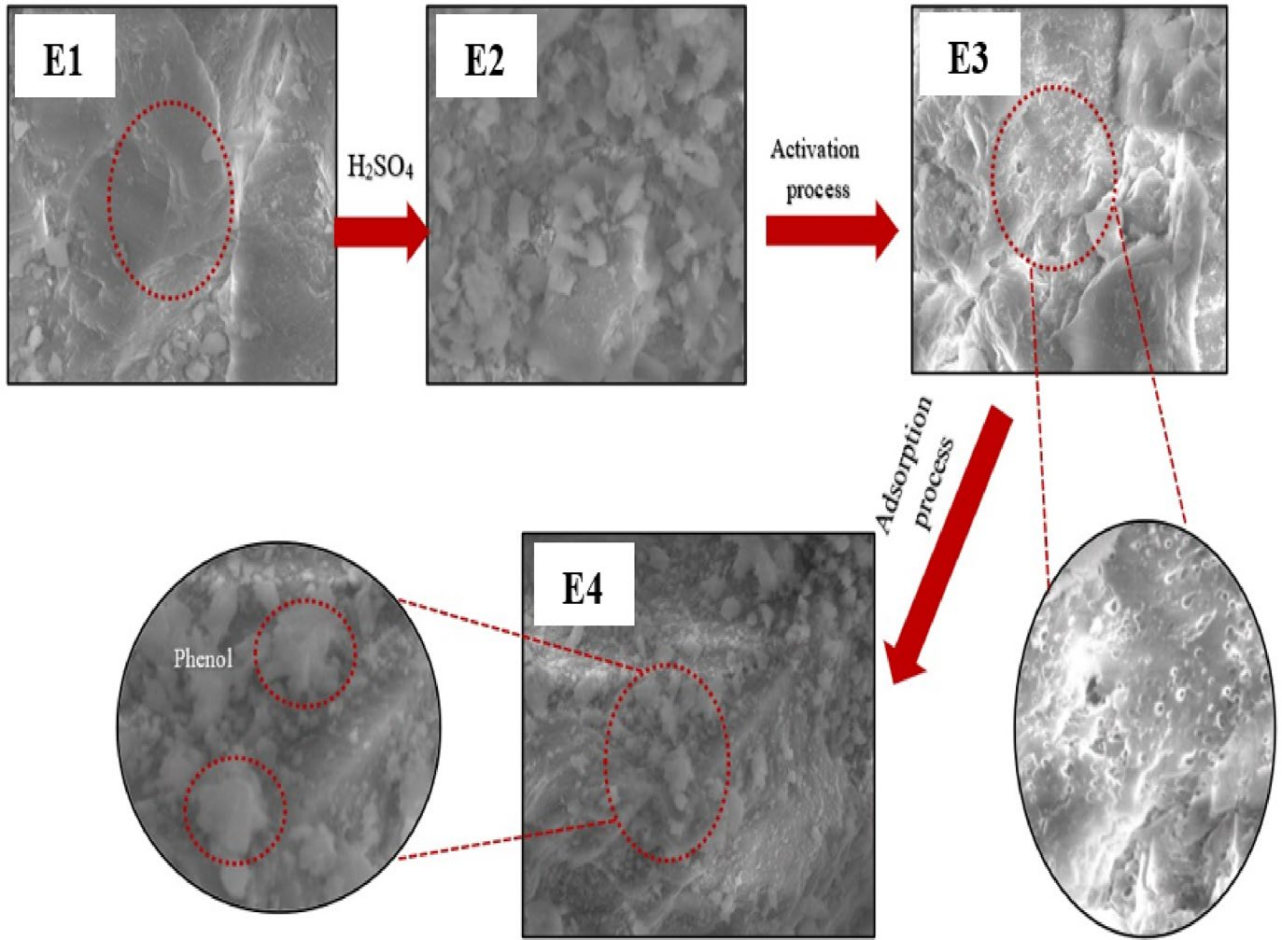
SEM image of: E1 Raw eggshell; E2 Pre-treated eggshell with H_2_SO_4_; E3 ACES before adsorption; and E4 ACES after adsorption.

Morphological changes in eggshell samples from E1 to E4 were examined via scanning electron microscopy (SEM) images. Eggshells possess a complex poly-porous structure, characteristic of a semi-permeable bio-membrane^32^, as evident in the SEM images (Fig. 5). Initially, crushed eggshell crystallites exhibited an irregular surface structure with pre-existing pores that allowed efficient impregnation, thereby increasing carbon content. The SEM image of E1 shows a textured surface with potential porosity, with a red dashed circle indicating a specific area of interest on the material’s surface. In E2, the surface morphology changes significantly, appearing more fragmented, possibly due to increased surface roughness or the formation of smaller particles, likely a result of acid treatment that altered the material’s structure.

Carbonization at around 650 °C resulted in a rougher morphology, enhancing surface area and adsorption potential compared to raw eggshells. The highly porous surface was observed in the SEM images of E3, indicating a transformation from CaCO_3_ to CaO with changes in crystallography, consistent with the literature^55,58,97^. The SEM image in (E3) reveals a notable change in surface morphology compared to (E2). The surface in E3 shows numerous pores and a larger surface area, with a red dashed circle highlighting a porous region. A zoomed-in view provides a closer look at these pores.

In E4, SEM images elucidate the phenol adsorption process, showing a film coating the active sites on the adsorbent surface, with phenol partially infiltrating the pores^60,98^, as confirmed by SEM imagery. Compared to E3, the SEM image of E4 shows slight filling or coating of the pores, suggesting phenol adsorption within the pores of the material. Red dashed circles highlight areas where adsorbed phenol may be present, and an inset visualizes phenol molecules interacting with the porous structure. Subsequently, the adsorbent’s surface accumulates metallic particles due to phenol adsorption, displaying a uniform distribution with irregular-shaped structures. Surface morphology analysis indicates that smaller particles offer a larger surface area for exchange, as confirmed by BET surface measurements. SEM images reveal particles with varying shapes and sizes, including pores, cavities, and channels, which serve as potential adsorption sites for pollutants^99^.

#### Transmission electron microscope (TEM)

**Fig. 6:**
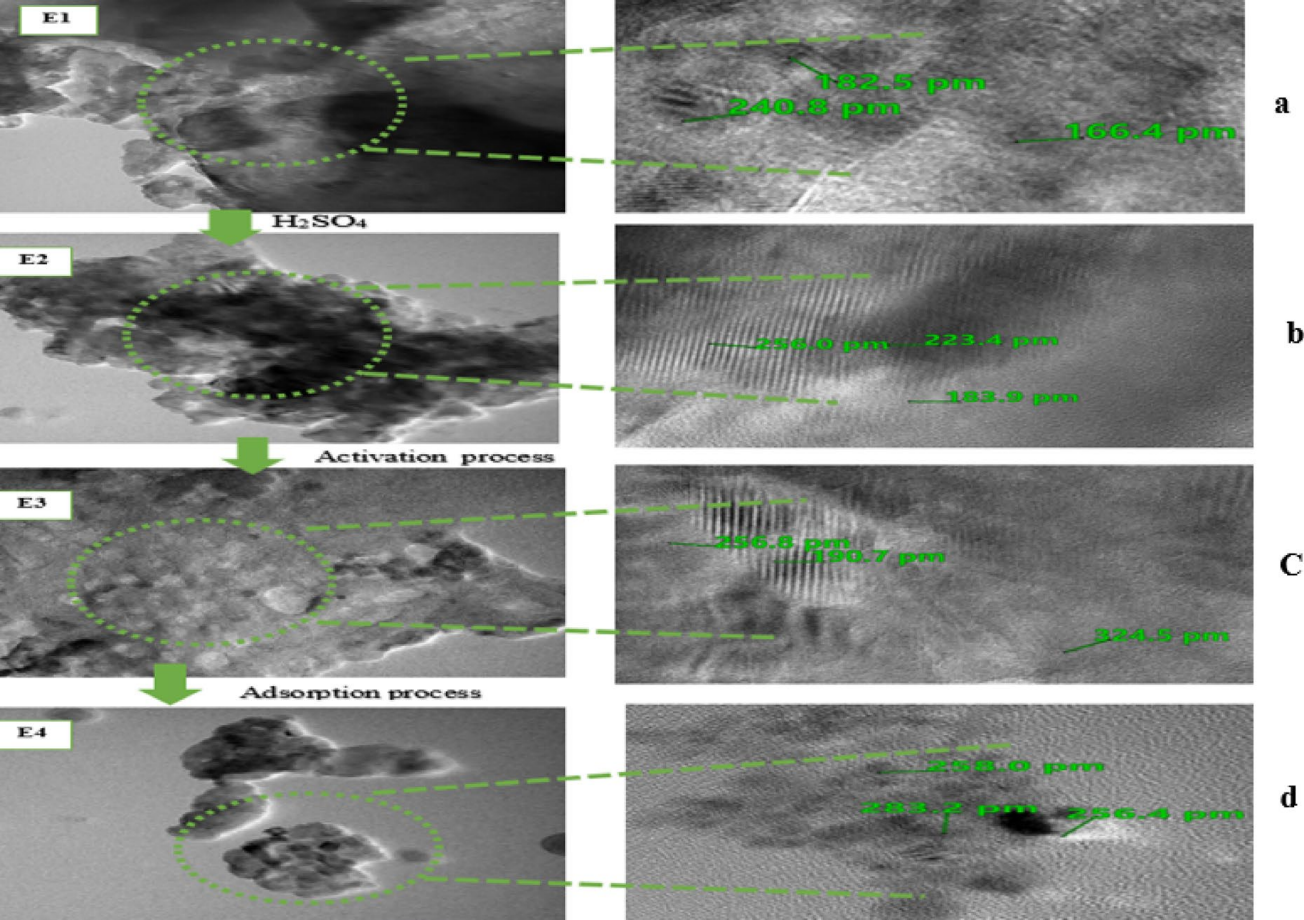
Transmission electron micrographs (TEM) of: (**a**) E1 Raw eggshell; (**b**) E2 Pre-treated eggshell with H_2_SO_4_; (**c**) E3 ACES before adsorption; and (**d**) E4 ACES after adsorption.

Transmission Electron Microscope (TEM) analysis was employed to assess the adsorbent’s structure^60^. TEM characterization was performed on raw eggshell (E1), eggshell treated with H_2_SO_4_ (E2), and ACES before and after phenol adsorption (E3 and E4), and the internal microporous network is displayed in Fig. 6.

Figure 6a, E_2_ reveals that the surface structure of the eggshell underwent alterations after pretreatment. The untreated eggshell had a regular and compact surface (Fig. 6b, E_1_). In contrast, pretreatment with H_2_SO_4_ induced significant alterations, resulting in a more porous and loosely packed structure with noticeable hollows, cracks, and finer particulates. This transformation contributed to a finer particle size distribution. While the pretreated eggshell surface appeared flatter, rougher, and more porous, the overall integrity of the raw material remained largely intact^60^.

Carbonizing pretreated eggshells exhibited enhanced porosity compared to their non-carbonized counterparts, as evidenced by the irregular cavities and holes depicted in Fig. 6C, E_3_. This enhanced porosity facilitates electrolyte ion adsorption by offering channels for forming an efficient electrical dual layer and enhancing charge accumulation. Moreover, it promotes rapid ion transfer and diffusion, as illustrated in Fig. 6d, E_4_. During carbonization, phenol was swiftly transported and adsorbed into the adsorbent’s inner pores due to the well-developed pore structure^60,100,101^.

#### Energy dispersive X-Ray (EDX) analysis

**Fig. 7:**
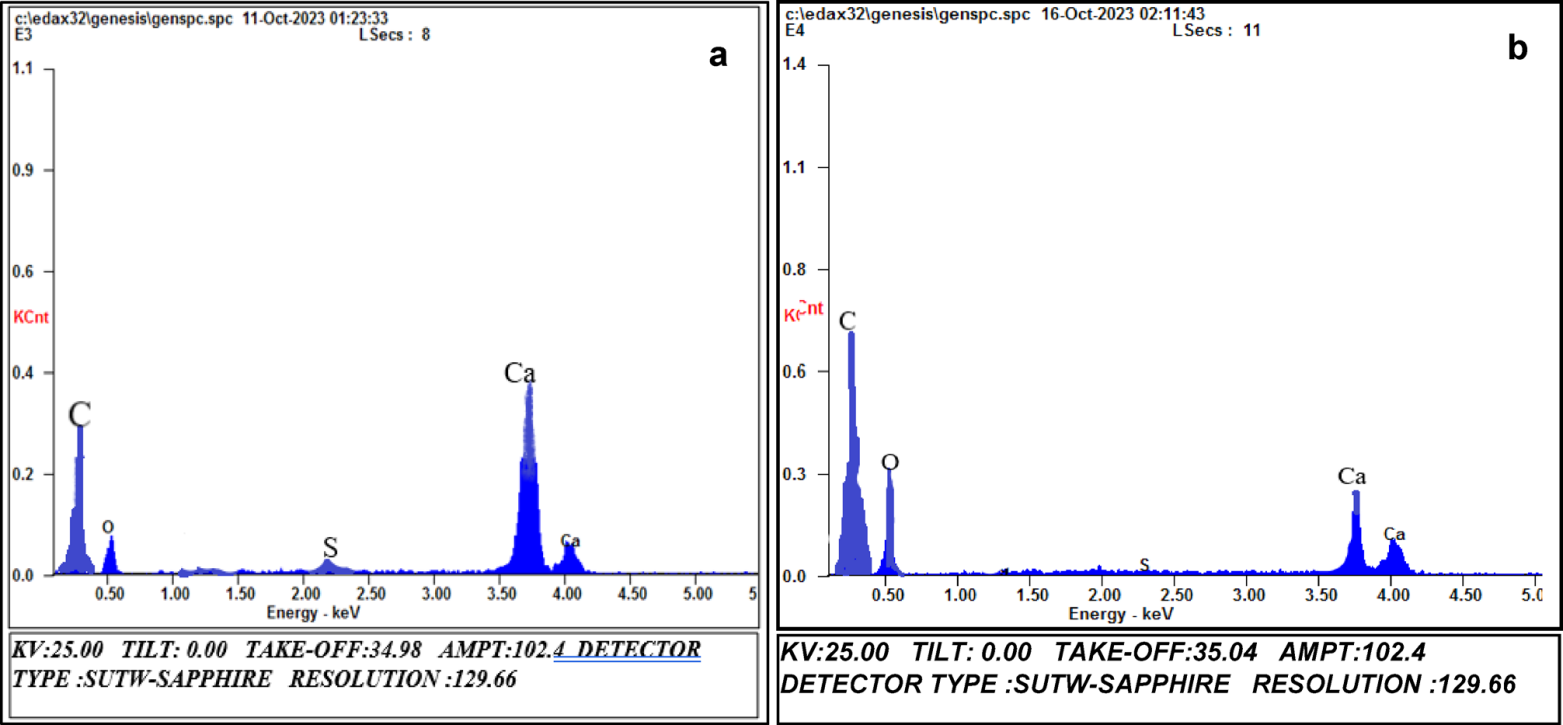
Electron Dispersion Spectrum (EDX) of ACES: (**a**) E3 before adsorption and (**b**) E4 after adsorption.

Energy Dispersive X-ray (EDX) analysis, depicted in Fig. 7; Table 2, confirmed the presence of carbon, oxygen, and calcium elements in the adsorbent. This finding is consistent with the composition of chicken eggshell, which is predominantly composed of 95–97% (CaCO_3_)^32,102,103^. Notably, the carbon existence further reinforces the appropriateness of eggshells as a precursor for activated carbon production^104^. Traces of sulphur (S) were detected in some samples owing to residual impregnating agent (H_2_SO_4_). Thorough washing with distilled water can eliminate these traces.

**Table 2:** EDX quantitative findings for ACES before and after the adsorption process.

Elements	Weight (%)		Atomic (%)	
	ACES before adsorption, E3	ACES after adsorption, E4	ACES before adsorption, E3	ACES after adsorption, E4
CK	40.97	50.55	25.99	30.87
OK	19.80	28.12	46.82	47.88
SK	00.63	00.26	00.42	00.29
CaK	37.51	21.07	26.77	20.96

#### X-ray diffraction analysis (XRD)

**Fig. 8:**
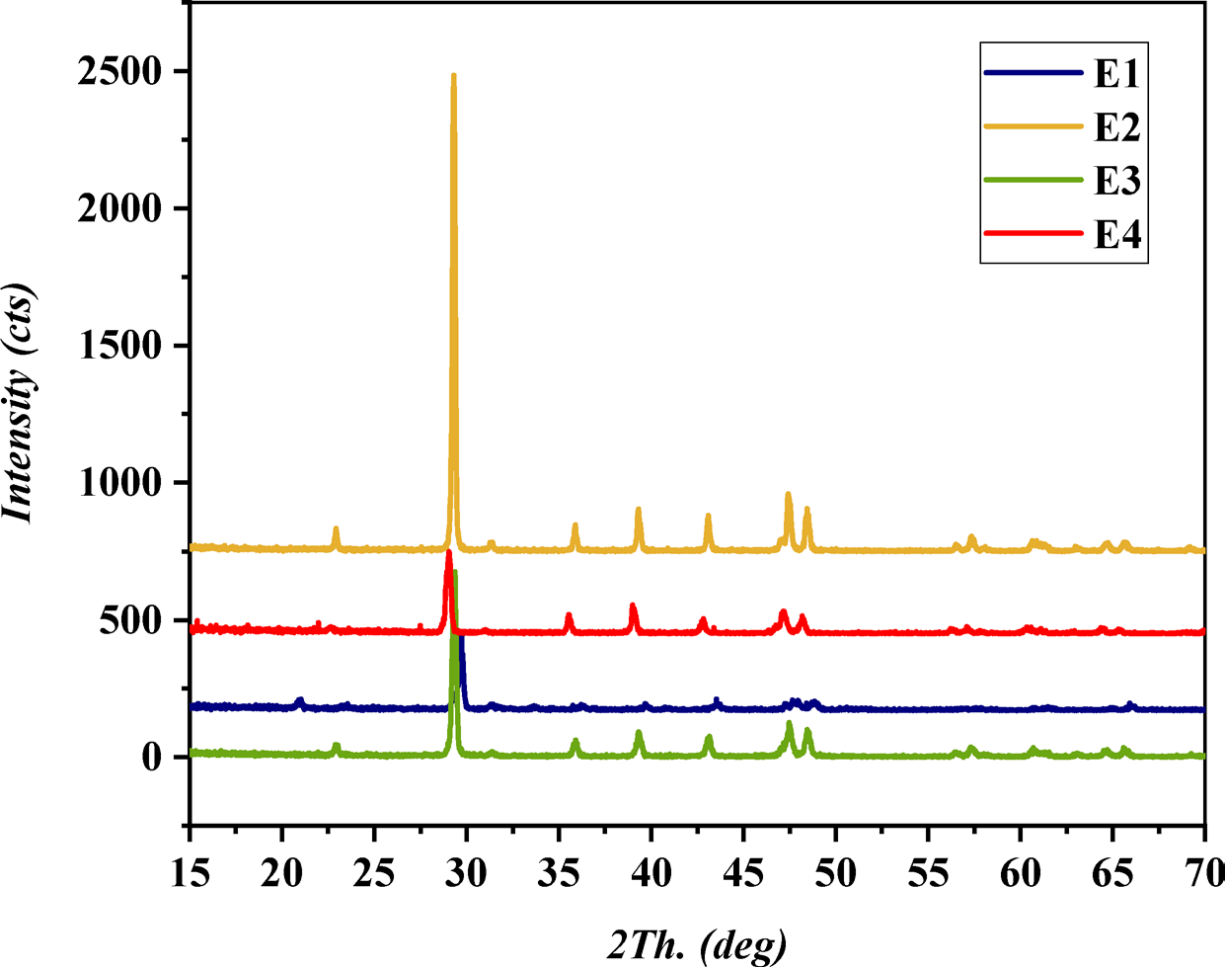
XRD analysis of eggshell: (E1) raw material, (E2) Pretreatment with H_2_SO4, (E3) ACES at 650^◦^C for 3 h before adsorption, and (E4) ACES after adsorption.

Figure 8 exhibits XRD analysis results for various stages of eggshell treatment and adsorption. Raw eggshell composition: In the raw eggshell (E1), prominent XRD peaks at 29.3°, 39.3°, 43.1°, 47.5°, and 57.2° 2θ correspond to the (104), (113), (202), (018), and (122) planes of crystalline calcite CaCO_3_, indicating its dominance in raw eggshells^73,105,106^.

Effects of pretreatment: Following sulfuric acid pretreatment (E2), the CaCO_3_ peaks significantly decrease in intensity or vanish, reflecting calcium carbonate dissolution and successful calcite removal. New Peaks After Pretreatment (E2): Small new peaks at 11.8°, 20.7°, 31.3°, 33.6°, and 40.8° 2θ could be related to sulphate phases like anhydrite, gypsum, or bassanite, or insoluble components like Ca(OH)_2_^79,91^, albeit with low intensity, indicating only trace levels of CaSO_4_ are formed during the pretreatment stage. These minor gypsum-associated peaks disappear after carbonization to CaO, indicating their transient nature^107^.

Carbonization Process: Post-carbonization, the pretreated eggshell (E3) exhibits intense peaks, including CaCO_3_, CaO, and CaS at 29°, 35.5°, 39°, 42.8°, 47.1°, 48.2°, and 67.7° 2θ. This confirms partial conversion of CaCO_3_ to CaO and CaSO_4_ to CaS at 650 °C, where CaSO_4_ peaks vanish^108,109^. CaSO_4_ appears only as an intermediate minor component during the pretreatment stage, as in the chemical reaction 2 ^110,111^.

Adsorption of Phenol: E4 peaks intensify after phenol adsorption, suggesting preferred orientation of CaCO_3_, CaCO_4_, CaO, and CaS crystals on the surface due to phenol interactions. Minor peaks at 43.0°, 35.8°, 47.4°, and 77.1° indicate possible CaS traces. Shifts in relative peak intensities suggest changes in Ca salt crystal orientation during adsorption, while the main phases of CaO and CaCO_3_ remain unchanged. This indicates that phenol interacts with the eggshell surface without altering the bulk crystal structure Fig. 8, Phenol adsorption mechanism. The XRD analysis offers valuable insights into the composition and crystal structure changes in eggshells during pretreatment, carbonization, and phenol adsorption, affirming the creation of suitable adsorbent sites for phenol adsorption following the removal of calcium salts and the conversion to calcium oxide and calcium sulphide at 650 °C^73,112^. The crystalline size can be determined from XRD data using the Scherrer equation, expressed as^113^:

3 $$D=K\lambda / B \text{ Cos }\theta$$

This approach is used to calculate the average crystallite size. In the equation, D represents the average crystallite size in nanometers (nm), K is the Scherrer constant (0.94), λ is the X-ray wavelength (for CuK, λ = 1.5406 Å), β is the full width at half maximum (FWHM) of the line in radians, and θ is the Bragg angle, which is half of 2θ. For this study, the values of β and D for the samples depicted in Table 3.

**Table 3:** XRD data (β and D values for the different samples).

	E1	E2	E3	E4
β	0.11	0.17185	0.25308	0.22559
D, nm	780.42	499.06	338.93	379.95

The diffraction peak width (FWHM) is inversely related to the crystallite size. A broader peak corresponds to smaller crystallites, while a sharper peak indicates larger crystallites. This relationship is evident in the calculated crystallite sizes: E1, with the narrowest FWHM (0.11 radians), has the largest crystallite size (780.42 nm), whereas E3, with a wider FWHM (0.25308 radians), has a smaller crystallite size (338.93 nm). These values suggest that the materials in these samples exhibit different average crystalline region sizes.

- **Sample E1** shows the largest crystallite size, indicating a higher degree of crystallinity or larger, more organized domains.
- **Samples E3 and E4** display smaller crystallite sizes, suggesting a less ordered structure, a higher defect density, or a different growth mechanism that results in smaller crystalline domains.
- **Sample E2** falls between E1’s larger size and the smaller sizes of E3 and E4.

These variations in crystallite size can significantly influence the physical and chemical properties of the materials. For example, smaller crystallites typically result in a larger specific surface area, which can enhance applications such as adsorption.

Acid-treated eggshells (E2) generally have smaller crystallite sizes than raw eggshells (E1), as the acid disrupts the calcium carbonate structure. In contrast, raw eggshells retain a larger, more ordered structure. Activated carbon from eggshells (ACES, E3) usually has smaller crystallites than acid-treated eggshells (E2) due to the activation process, which involves heating to break down the structure into finer, porous material. Acid treatment removes impurities but does not significantly reduce crystallite size. The activation process reduces crystallite size by creating a more disordered, porous structure. During adsorption (e.g., phenol removal), interactions with the target substances may cause slight swelling or aggregation, increasing crystallite size and altering the surface morphology. As a result, ACES before adsorption (E3) has smaller crystallites than after adsorption (E4).

#### Zeta potential analysis

The phenol adsorption onto eggshell particles is a complex process involving electrostatic interactions, van der Waals forces, and hydrogen bonding. The zeta potential plays a crucial role in determining the strength of the electrostatic interactions between the eggshell particles and the phenol molecules. A higher negative charge on the eggshell particles leads to stronger electrostatic interactions, enhancing the adsorption process^114^.

**Fig. 9:**
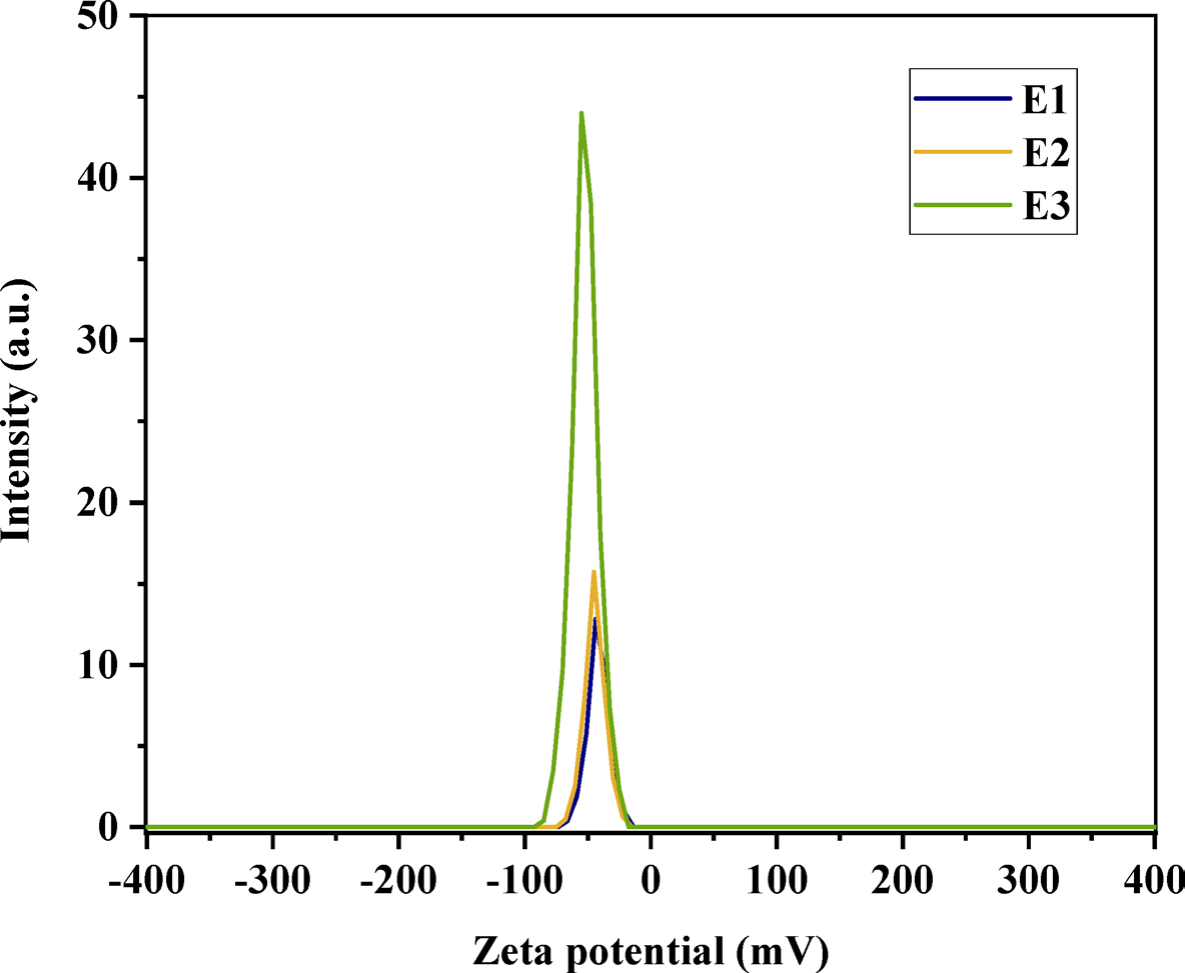
Zeta potential distribution of: E1 raw eggshell; E2 Pretreated eggshell; and E3 ACES.

Based on the zeta potential (ZP) values depicted in Fig. 9, ACES (E3) is the most effective material for adsorbing phenol. This is because E3 has the lowest ZP value (− 52.4 mV), which means that it has the strongest negative charge. Phenol is also negatively charged, so it will be attracted to and adsorbed onto the surface of E3.

Raw eggshell (E1) has the highest ZP value, which means that it has the weakest negative charge. This means that phenol will be less attracted to and adsorbed onto the surface of E1.

Eggshell treated with H_2_SO_4_ (E2) has a ZP value (-44.6 mV) that is slightly lower than that of E1 (-41.9 mV). This means that E2 will be slightly more effective at adsorbing phenol than E1. Overall, the ZP values suggest that E3 is the most effective material for adsorbing phenol, followed by E2 and then E1.

### Adsorption process enhancement via RSM

Employing the RSM-CCD statistical design, twenty-nine experiments were conducted, encompassing the specified ranges outlined in section (Optimization and Modelling). Equation (4) reveals the mathematical correlations of phenol removal response (Y), considering the interaction effects of operational parameters and derived from the experimental results in Table 4. The complete experimental dataset was optimally fitted to a reduced cubic model, as it yielded the highest predicted R^2^ and adjusted R^2^ values (Table 5).

**Table 4:** Experimental percentage and predicted percentage of phenol removal efficiency.

Run	A: pH	B: Initial conc.	C: Temp	D: Dose	Elimination% observed, Y	Elimination % predicted
Unit	–	mg/L	degree Celsius	gm	%	%
1	6	62.5	50	3	79.3	80.43
2	9	100	75	3	74.69	76.83
3	6	62.5	50	3	79.4	80.43
4	6	100	50	3	71.81	72.07
5	6	25	25	4	95.65	95.91
6	6	62.5	50	3	79.26	80.43
7	6	62.5	50	3	79.34	80.43
8	6	62.5	50	3	79.4	80.43
9	3	25	25	4	99.01	98.34
10	9	25	25	4	87	89.08
11	3	25	75	1	80.64	81.37
12	3	25	75	5	89.5	89.93
13	9	100	75	5	84.37	83.87
14	6	62.5	50	3	79.39	80.43
15	3	25	25	1	86.64	89.41
16	9	25	75	1	77.61	78.85
17	3	100	75	5	87.03	87.50
18	6	62.5	25	3	85.23	85.37
19	3	62.5	50	3	86.73	85.22
20	3	100	25	5	90.2	92.01
21	6	62.5	50	1	76.31	74.77
22	9	25	25	1	80.46	78.52
23	9	25	75	5	92.48	92.94
24	3	100	75	1	78.32	78.94
25	3	100	25	1	85.6	83.45
26	9	100	25	1	76.83	78.20
27	9	62.5	50	3	84.7	81.02
28	9	100	25	5	93.44	92.28
29	6	62.5	75	3	85.79	80.21

4 $$\begin{gathered} {{Y}}\% = {{88}}.{{52}} + {{6}}.{{66}}{{A}} + {{0}}.{{31}}{{B}}{-}{{0}}.{{69}}{{C}} + {{1}}.{{45}}{{D}}{-}{{0}}.{{19}}{{AB}} + {{0}}.{{042}}{{AC}} \hfill \\ \quad + {{0}}.{{23}}{{AD}} + {{0}}.{{003}}{{BC}}{-}{{0}}.{{84}}{{A}}^{{{2}}} + {{0}}.{{004}}{{C}}^{{{2}}} - {{0}}.{{0005}}{{ABC}} + {{0}}.{{02}}{{A}}^{{{2}}} {{C}} \hfill \\ \end{gathered}$$

Experimental results were analysed using RSM in conjunction with CCD statistical software to approximate dependent variable responses and determine optimal operating conditions. Statistical significance was assessed through analysis of variance (ANOVA), as detailed in Table 5. The ANOVA table breaks down the variability of phenol removal (%) into contributions from different factors, with P-values indicating the significance of each factor. P-values below 0.0500 signify the significance of model terms. In this instance, A, B, C, D, ABC, and A²B have statistically significant effects on phenol removal (%) at a 95.0% confidence level. The correlation coefficient (R^2^ of 91.55% illustrates the model’s strong fit to the experimental data.

**Table 5:** ANOVA test results for the response surface reduced quartic model for phenol adsorption.

Source	Sum of squares	DF	Mean square	F-value	*p*-value	
**Model**	1099.04	12	91.59	14.45	< 0.0001	Significant
A: pH	78.86	1	78.86	12.44	0.0028	
B: Initial conc	116.39	1	116.39	18.36	0.0006	
C: Temp	120.12	1	120.12	18.95	0.0005	
D: Dose	439.84	1	439.84	69.39	< 0.0001	
AB	0.2437	1	0.2437	0.0384	0.8470	
AC	4.89	1	4.89	0.7710	0.3929	
AD	23.97	1	23.97	3.78	0.0696	
BC	6.90	1	6.90	1.09	0.3121	
BD	28.04	1	28.04	4.42	0.0516	
CD	19.13	1	19.13	3.02	0.1016	
A²	37.53	1	37.53	5.92	0.0271	
B²	56.33	1	56.33	8.89	0.0088	
C²	101.42	16	6.34			
D²	101.40	11	9.22	2666.82	< 0.0001	
ABC	0.0173	5	0.0035			
ABD	1200.46	28				
ACD	1099.04	12	91.59	14.45	< 0.0001	
BCD	78.86	1	78.86	12.44	0.0028	
ABCD	116.39	1	116.39	18.36	0.0006	
**Residual**	120.12	1	120.12	18.95	0.0005	
Lack of Fit	439.84	1	439.84	69.39	< 0.0001	Significant
Pure Error	0.2437	1	0.2437	0.0384	0.8470	
**Cor Total**	4.89	1	4.89	0.7710	0.3929	
**Standard deviation**	2.52					
**Mean**	83.66					
**R²**	0.9155					
**Adjusted R²**	0.8522					
**Predicted R²**	0.6885					
**C.V. %**	14.1428					
**Adequate precision**	0.8522					

*DF: Degree of Freedom **CV: Coefficient of Variation.

**Fig. 10:**
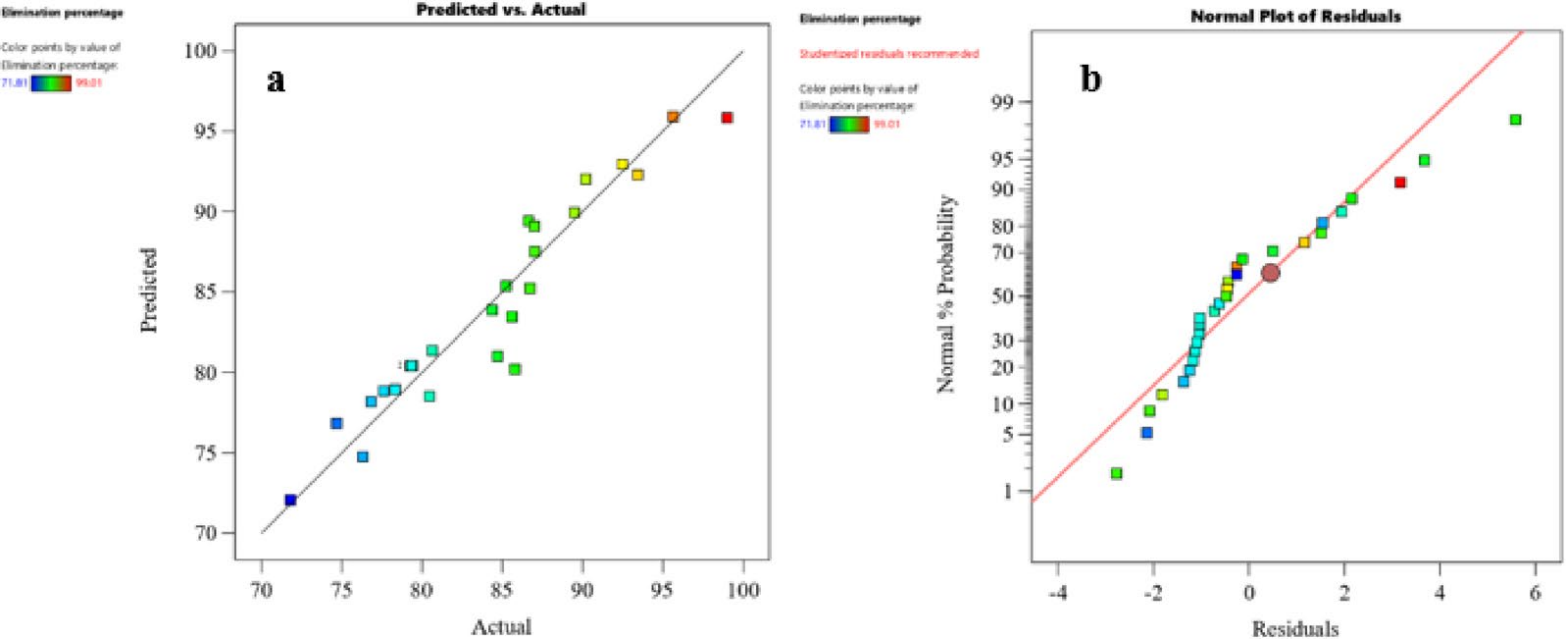
Assess the adequacy of the model used the plot of the residuals: (**a**) Predicted versus actual values and (**b**) Normal probability of residuals.

**Fig. 11:**
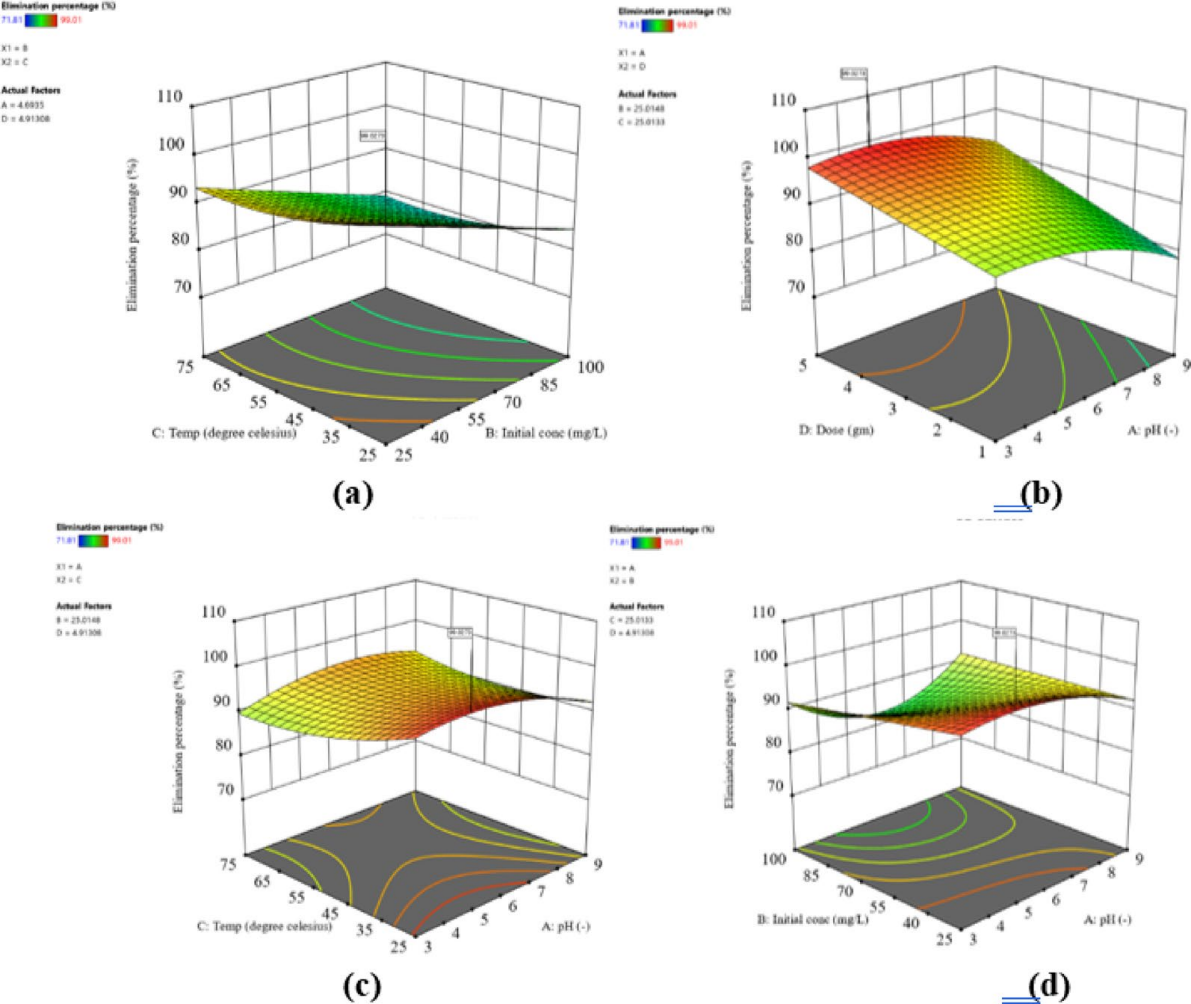
3D response surface, exhibiting the impact of pH, initial ion concentration, temperature, and ACES dosage on the phenol percentage elimination.

The predicted values generated by the reduced cubic model were evaluated against the experimental data to gauge the model’s effectiveness. Figure 10a displays the agreement between experimental and predicted phenol removal percentages, affirming the significance of the models. The normal probability plot of residuals in Fig. 10b shows a reasonably straight line, indicating the model’s appropriateness. The 3D response surface plots (Fig. 11a-d) illustrate the interaction effects between variables. Figure 11a reveals a simultaneous change in temperature and initial phenol concentration, resulting in a decrease in removal percentage. Figure 11b demonstrates the influence of pH and ACES dose on phenol adsorption, with increasing ACES dose leading to an increased removal percentage. Additionally, response surface plots were generated for pH and temperature variation (Fig. 11c), and initial phenol concentration and pH variation (Fig. 11d).

**Fig. 12:**
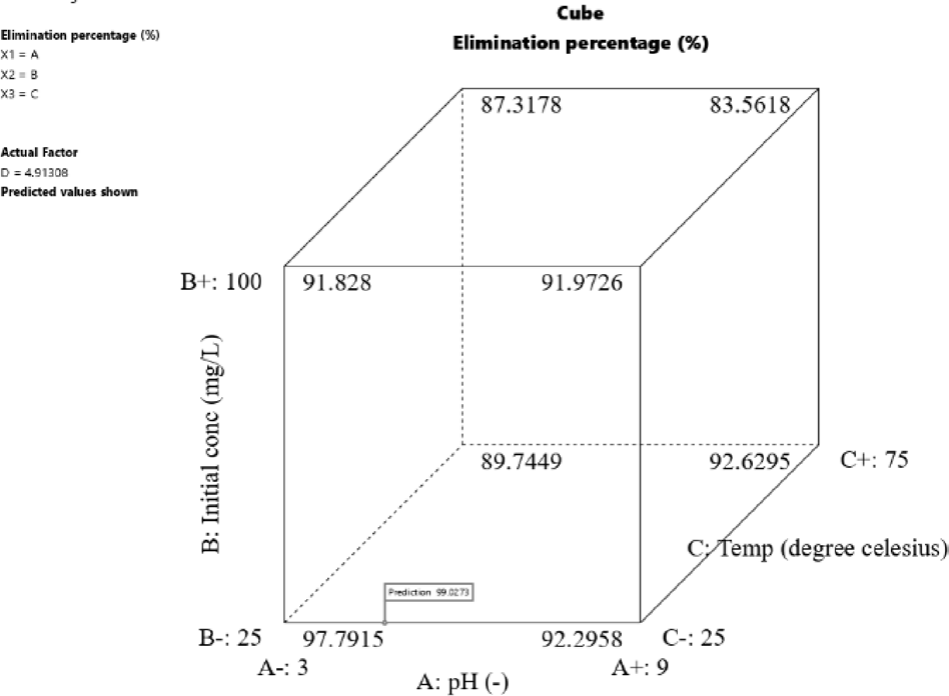
Cube response surface, exhibiting the impact of pH, initial ion concentration, and temperature on the phenol percentage elimination.

**Fig. 13:**
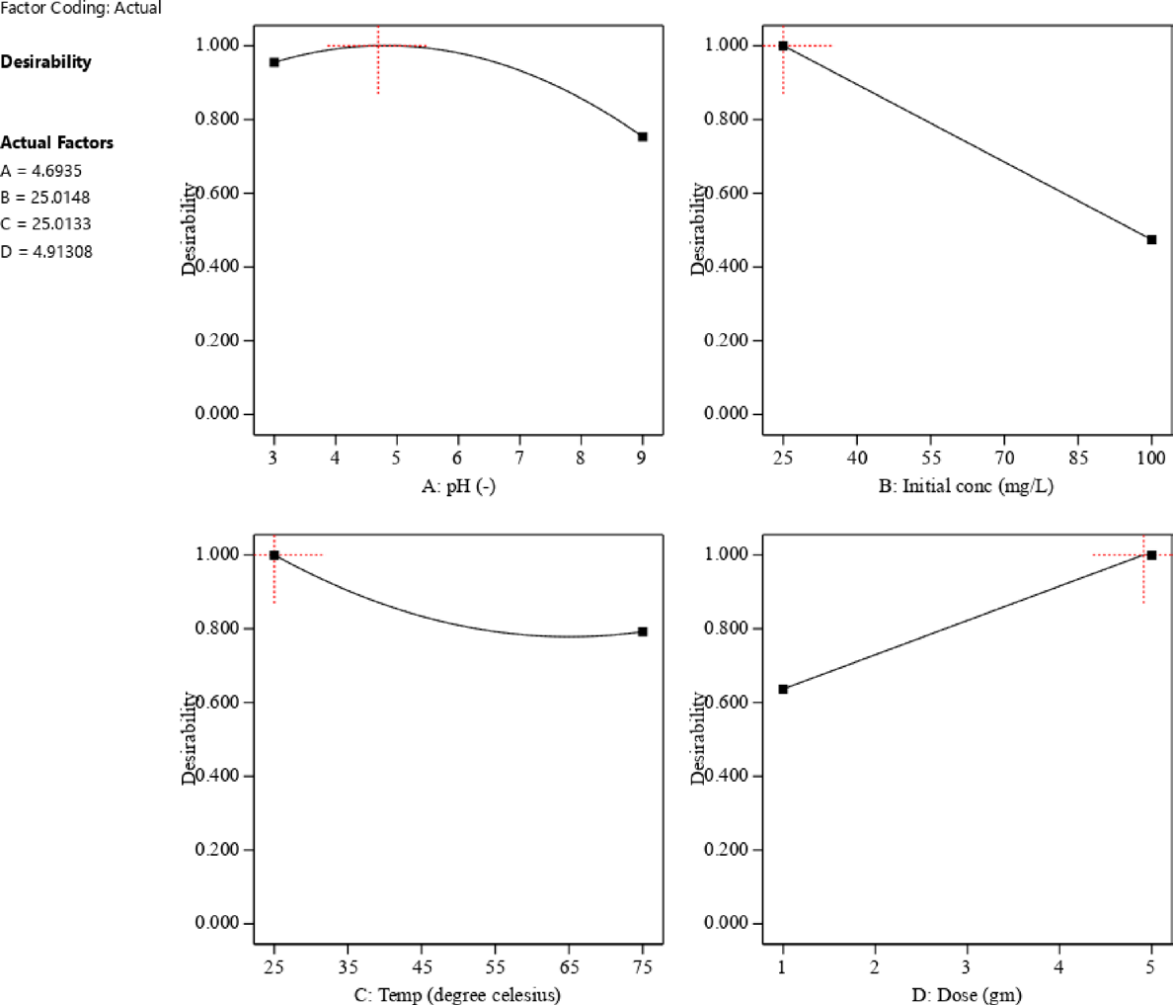
Desirability ramp of phenol adsorption by ACES.

**Fig. 14:**
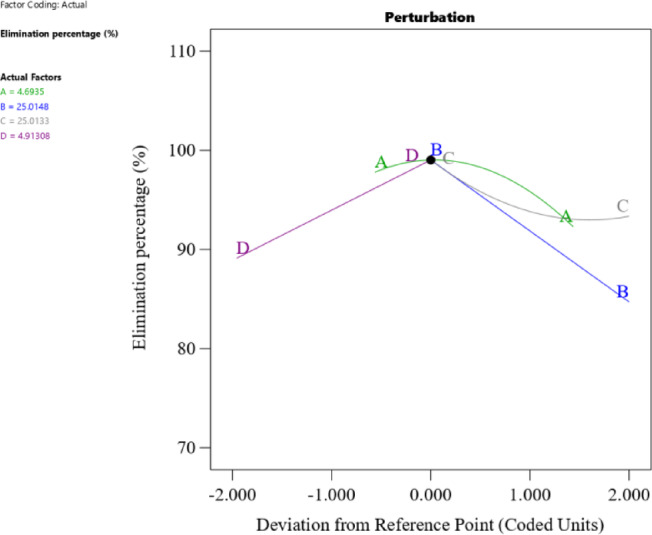
Perturbation plot of phenol adsorption by ACES.

The cube response surface plot (Fig. 12) illustrates the interaction effects between variables. Optimization revealed that the optimum conditions for phenol removal were an initial phenol concentration of 25.015 ppm, ACES dose of 4.913 gm, pH of 4.693, and temperature of 25.013 °C for 80 min (Fig. 13), yielding a reasonable removal percentage of 99.87%. The experimental result closely matched the predicted value of 99.63%, confirming the validity of the models. The perturbation plot in Fig. 14 highlights the most influential operating variables: ACES dose, temperature, pH, and initial phenol concentration, in descending order of importance.

### Impact of operating factors on adsorption competence

**Fig. 15:**
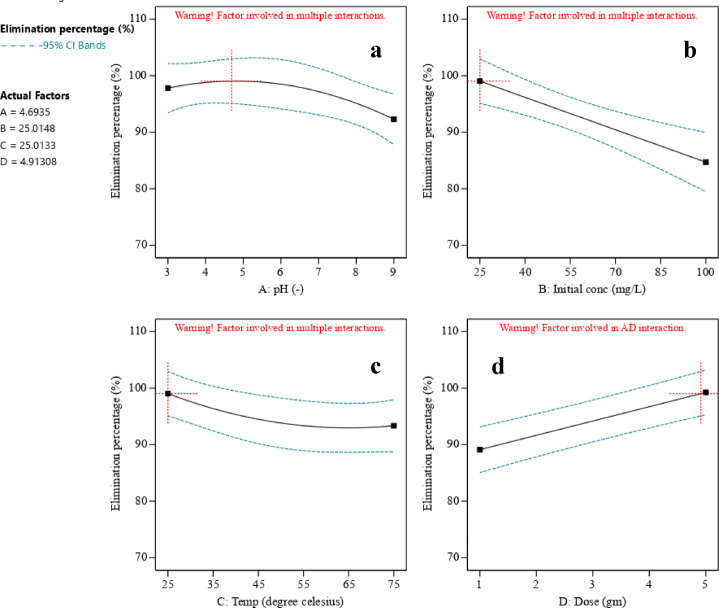
Impact of operating factors on phenol removal: (**a**) pH, (**b**) initial concentration, (**c**) temperature, and (**d**) ACES dose.

Figure 15 showcases the three-dimensional relationships between phenol adsorption and diverse influencing factors, including temperature, initial phenol concentration, ACES dosage, and solution pH.

Varying the initial pH (Fig. 15a) from 3 to 9 resulted in a decrease in mean phenol removal after pH 4.693. The pH significantly influences phenol removal, with the best results achieved at pH 4.693, attributed to phenol ionization and surface charge. As a weak acid, phenol dissociates into phenoxide ions. Owing to favourable factors such as a higher exchangeable ion rate and a more suitable adsorbent^58,115^, the optimal pH for attaining the desired phenol removal standard is defined to be between 3 and 4. Notably, phenol removal decreases in alkaline conditions, possibly because of increased turbidity, which aligns with observations reported by Daraei et al.^55^, who noted the fastest removal rate at pH 3.5. Similar trends were observed in other studies^17,116^. The pH of the point of zero charge (pH_ZPC_), representing the neutral charge, is crucial, as pH above it reduces phenol sorption due to electrostatic repulsion and competition with hydroxides^58^.

Conversely, an increase in initial phenol concentration (Fig. 15b) led to a decline in average phenol removal owing to hindered mass transfer resulting from the saturation of active adsorption sites by phenol molecules. Despite this, the enhanced concentration gradient served as a driving force, overcoming all mass transfer resistances between the liquid and solid phases for phenol, leading to improved equilibrium sorption until sorbent saturation was attained^58^. Furthermore, the pH_ZPC_ shifted with increasing phenol concentration, diminishing electrostatic attraction between the adsorbent and phenoxide ions.

Increasing temperature (Fig. 15c) resulted in a reduction in phenol removal percentage, attributed to decreased surface activity of the adsorbent at higher temperatures, which necessitates more energy for stability in the liquid medium, affecting the sorption balance^117^. The decline in adsorption can be clarified by the increased solubility at higher temperatures, leading to a reduction in adsorbate-adsorbent interactions. A comparable trend was observed by MDA Taufik et al.^118^. This outcome points to the exothermic nature of the system and its preference for lower temperatures, with the highest phenol removal observed at 298 K.

Next, the variable of interest, adsorbent dosage, influenced removal efficiency (Fig. 15d). Phenol removal increased with an ACES dosage increase from 1 to 4.913 gm. More adsorbent dosage enhanced surface area, sorption capacity, active adsorption sites, exchangeable sites, and porosity^58,115^. The most effective dosage was found to be 4.913 gm of ACES. This phenomenon likely arises from the exposure of previously unavailable adsorbent sites, enabling direct interaction between phenoxide ions in the water and the adsorbent surface, thereby enhancing adsorption capacity. However, increasing the adsorbent dosage beyond the optimal dosage will cause a decline in the surface area owing to the formation of aggregates, raising the pH^58^.

### Behaviour of phenol adsorption on ACES

Phenol adsorption from aqueous solutions using ACES depends on factors such as surface area, active functional groups, and cation exchange capacity. The interactions between ACES and pollutants involve both physical and chemical adsorption. ACES’s porous structure, rich in micropores and mesopores, aids in trapping pollutants, making it effective for phenol adsorption. Its large surface area provides ample adsorption sites for a variety of organic and inorganic compounds, including pollutants, heavy metals, and pharmaceuticals. Physical adsorption is driven by van der Waals forces, while electrostatic attraction, influenced by ionic strength and active sites, is stronger and involves interactions between charged species. Chemical adsorption also contributes, with functional groups on the ACES surface forming hydrogen bonds and dipole-dipole interactions with contaminants^119^.

The process of Phenol adsorption by ACES (E3) comprises four essential stages. First, a liquid film allows phenol molecules to diffuse into the porous ACES structure, which has a high surface area (diffusion process) due to the thermal activation process.

In the second step, at acidic pH, phenol takes on a macromolecular form while the mesoporous ACES framework provides abundant surface area. Here, phenol initially enters pores through pore filling. The large specific surface area contains functional groups serving as active sites that facilitate chemical bonding and electrostatic attraction to phenol, where the eggshell surface becomes positively charged, and phenol acquires a negative charge, resulting in electrostatic attractions that bring them closer^120^.

Third, immobilization occurs through van der Waals forces initially, followed by π-π stacking between the phenol’s aromatic ring and ACES’s delocalized electrons, adding further binding forces to the process^121^.

**Fig. 16:**
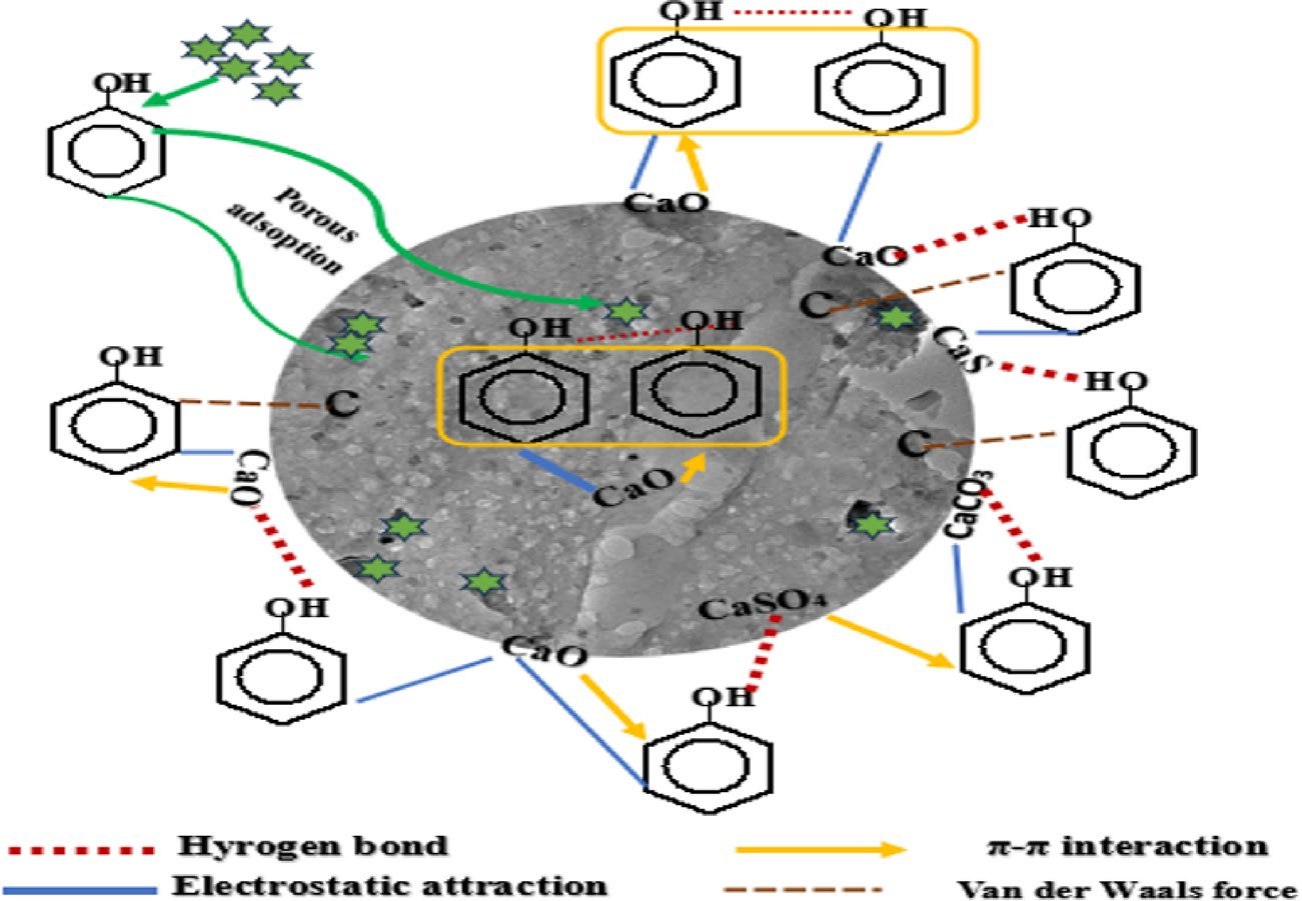
Phenol adsorption mechanism.

In the fourth step, the calcium oxide surface comes into play, containing reactive hydroxyl groups that can interact with phenol. Hydrogen bonding forms between the hydroxyl groups on the eggshell surface and those on phenol, effectively attracting and binding phenol molecules to the surface^122^. Figure 16 elucidates this phenol adsorption mechanism onto ACES. This mechanism suggests that the adsorption process involves a combination of electrostatic attraction, π-π interactions, and hydrogen bonding between the phenol molecules and the functional groups present on the surface of the activated carbon.

Delving into the mechanism of phenol adsorption necessitates pinpointing the dominant process governing its rate. In solid-liquid adsorption, the journey of the solute can be governed by external mass transfer, internal diffusion within the adsorbent particles, or a complex interplay of both.

To unveil the dominant factor, a well-established intraparticle diffusion model^60^ was utilized. This model sheds light on the diffusion mechanism when mass transfer dictates the pace of adsorption. The initial diffusion rate is described by an equation derived in a previous study^123^, as outlined in the model.

**Fig. 17:**
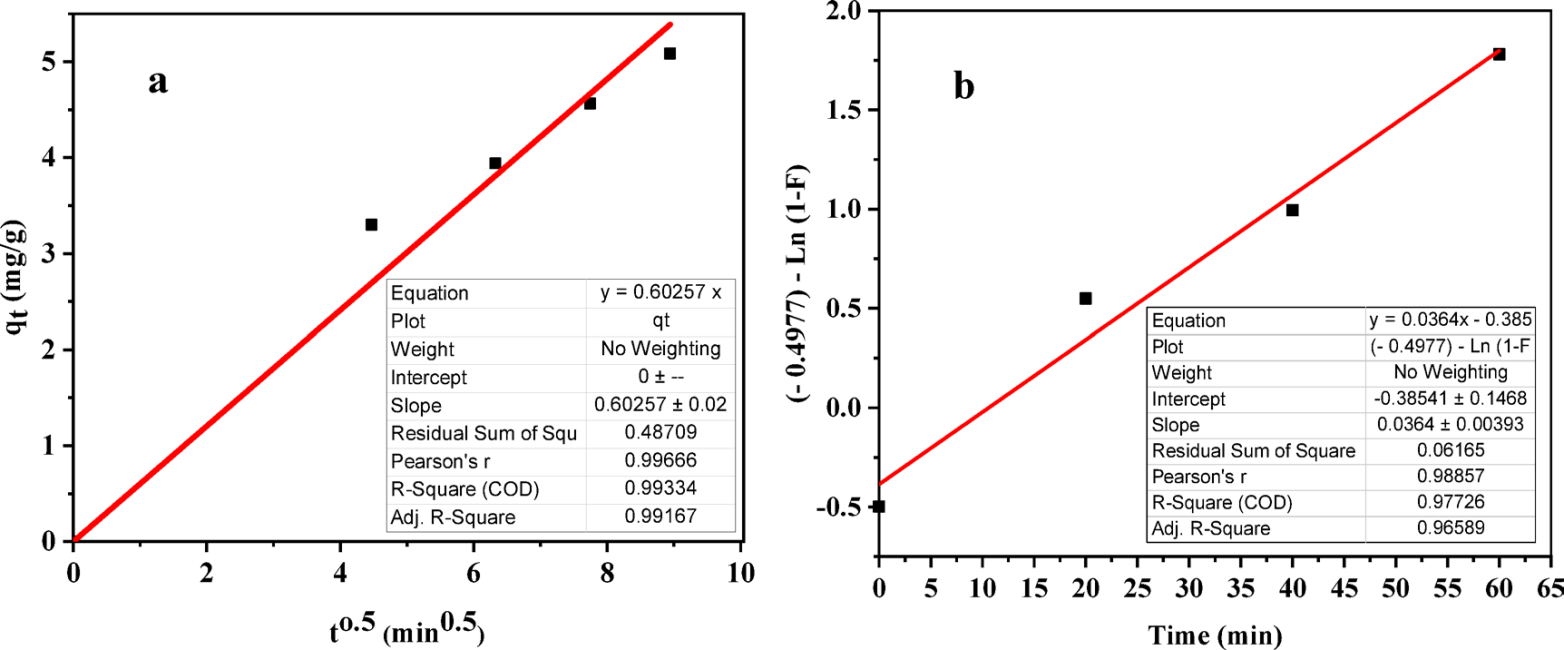
Rate-controlling step in phenol adsorption: (**a**) Intraparticle diffusion model, and (**b**) Boyd model.

Under ideal conditions, the intraparticle diffusion constant (k_d_) in mg/gm min^0.5^ was extracted from the slope of a specific plot (q_t_ vs. t^0.5^), as shown in Fig. 17a. The estimated k_d_ value of 0.60257 mg/gm min^0.5^ and a high regression coefficient (R^2^ = 99.33%) suggest a strong fit. Additionally, the linear nature of the plot with an origin intercept (Fig. 17a) indicates that intraparticle diffusion might be the sole factor controlling the adsorption rate.

To further refine the understanding of the true rate-limiting step, the Boyd model^124^ was applied to analyse the kinetic data. This model relies on a specific plot [– 0.4977 – ln (1 – F)] vs. t, where linearity differentiates between external mass transfer and intraparticle diffusion as the dominant influence^60,125^. In this plot, a straight line passing through the origin indicates intraparticle diffusion control, while deviations suggest external mass transfer control or film-diffusion control^124^. The straight line observed in Fig. 17b, deviating from the origin, suggests involvement of film diffusion in phenol adsorption onto ACES. Analysis revealed film diffusion, not intraparticle diffusion alone, controls phenol adsorption. Nevertheless, the contribution of other kinetic models cannot be entirely ruled out. Similar findings were obtained in the reported work^57^. The Boyd model parameters estimated from this plot provide additional insights (B = 0.0364, D = 2.076 × 10^−11^, R² = 97.73%).

### Adsorption kinetics of phenol

**Fig. 18:**
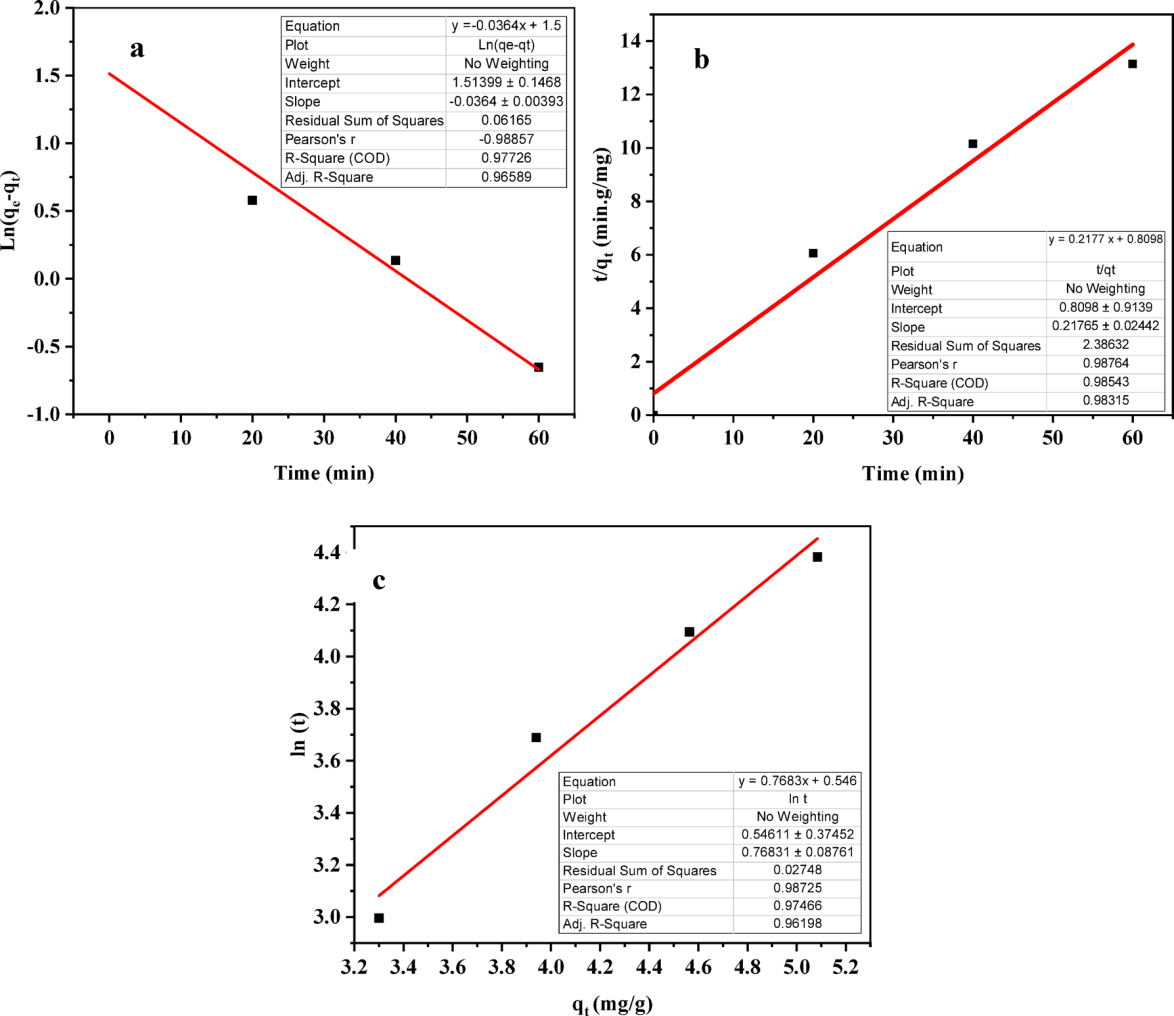
Adsorption kinetic model of phenol onto ACES: (**a**) pseudo 1st order model, (**b**) pseudo 2nd order model, and (**c**) Elovich model.

The adsorption kinetic run was performed at optimal factors (25.015 mg/L initial phenol concentration, a temperature of 25.013 °C, a pH of 4.693, and an adsorbent dosage of 4.913 gm for 80 min). Pseudo-first-order (Eq. 5), pseudo-second-order (Eq. 6), and Elovich (Eq. 7) kinetic models are used to analyse the kinetic data^60^. Phenol adsorption onto ACES favoured chemisorption (R²= 98.543%) over physical adsorption, as revealed by kinetic analysis (Fig. 18). This finding corroborates existing literature^17,66,126–129^ but diverges from outcomes announced by Mukherjee et al.^2^. The calculated q_e_ (4.6 mg/gm) closely matches with anticipated q_e_ (5.08 mg/gm), as elucidated in Table 6, further validating the chemisorption mechanism.

5 $${\text{Ln}}\left( {{{q}}_{{{e}}} - {{q}}_{{{t}}} } \right) = {{\ln}}\left( {{{q}}_{{{e}}} } \right){-}{{K}}_{{{1}}} {{t}}$$

6 $$\:\frac{{t}}{{{q}}_{{t}}}=\:\frac{1}{{{K}}_{2}\:.{{q}}_{{e}}2}+\:\frac{{t}}{{{q}}_{{e}}}$$

7 $${{q}}_{{{t}}} = {{ a }} + {{ b}}.{\text{Ln}}\left( {{{ t}}} \right)$$

Where, q_e_ and q_t_ represent the quantity of chromium ions adsorbed mg/gm) at equilibrium and at time t, individually, K_1_ (minute^− 1^) (0.0364 min^− 1^) and K_2_ (gm.min^− 1^.mg^− 1^) (0.0585 gm.min^− 1^.mg^− 1^) are the rate constants of pseudo-first-order and pseudo-second-order, respectively.

**Table 6:** Reaction rate of 25.015 ppm initial phenol concentration.

Reaction rate model	Rate constant	Adj. *R*^2^	*R*^2^ (COD)	RSS	Estimated q_e_, mg/gm	Expected q_e_, mg/gm
First order model	0.0364 min^− 1^	96.59%	97.726%	0.06165	4.54	5.08
Second order model	0.0585 gm.min^− 1^.mg^− 1^	98.315%	98.543%	2.38632	4.6	
Elovich model	-	96.198%	97.466%	0.02748	-	
Intraparticle diffusion model	0.060257 mg/gm.min^1/2^	99.17%	99.334%	0.48709	-	

### Adsorption isotherm of phenol

The Freundlich isotherm model assumes a heterogeneous adsorption system where the adsorbent surface is non-uniform. In contrast, the Langmuir isotherm model postulates a homogeneous surface with a finite number of identical adsorption sites. The Temkin isotherm is more suitable for describing adsorption energy distribution on heterogeneous surfaces, while the Dubinin-Radushkevich isotherm is an empirical model that accounts for both homogeneous and heterogeneous adsorption processes^127^. The Langmuir, Freundlich, Dubinin-Radushkevich, and Temkin isotherm models are represented by Eqs. 8–11, respectively:

8 $$\:\frac{{Ce}}{{qe}}=\:\frac{1}{{qm}.{KL}}+\:\frac{{Ce}}{{qm}}$$

9 $${\text{Log }}q_{{{e}}} = {\text{ log }}K_{{{f}}} + {{ 1}}/n{\text{ log }}C_{{{e}}}$$

10 $${\text{ln}}q_{{{e}}} = {\text{ ln}}q_{{{m}}} - {{ K}}_{{{\text{DR}}}} .\varepsilon ^{{{2}}}$$

11 $${{q}}_{{{e}}} = {B \text{ lnK}}_{{{t}}} + {B \text{ lnC}}_{{{e}}}$$

12 $${{\varepsilon }} = {\text{RT ln}}\left( {{{1}} + \frac{1}{{C_e}}} \right)$$

Where C_e_ (mg/L) represents the equilibrium concentration of phenol, and q_e_ (mg/gm) denotes the quantity of phenol adsorbed per gram of activated carbon at equilibrium. The Freundlich model constants, K_f_ and n, characterize adsorption capacity and intensity, respectively. In Eq. 9, B is derived from the relationship B = RT/b_t_, where R is the gas constant (8.314 J/mol. K), T (°K) is the absolute temperature, b is the Temkin energy constant associated with the heat of adsorption (J/mol), and K_t_ is the Temkin isotherm constant (L/gm). K_DR_ is a constant linked to the mean free energy of adsorption (mol^2^/K.J^2^).

**Fig. 19:**
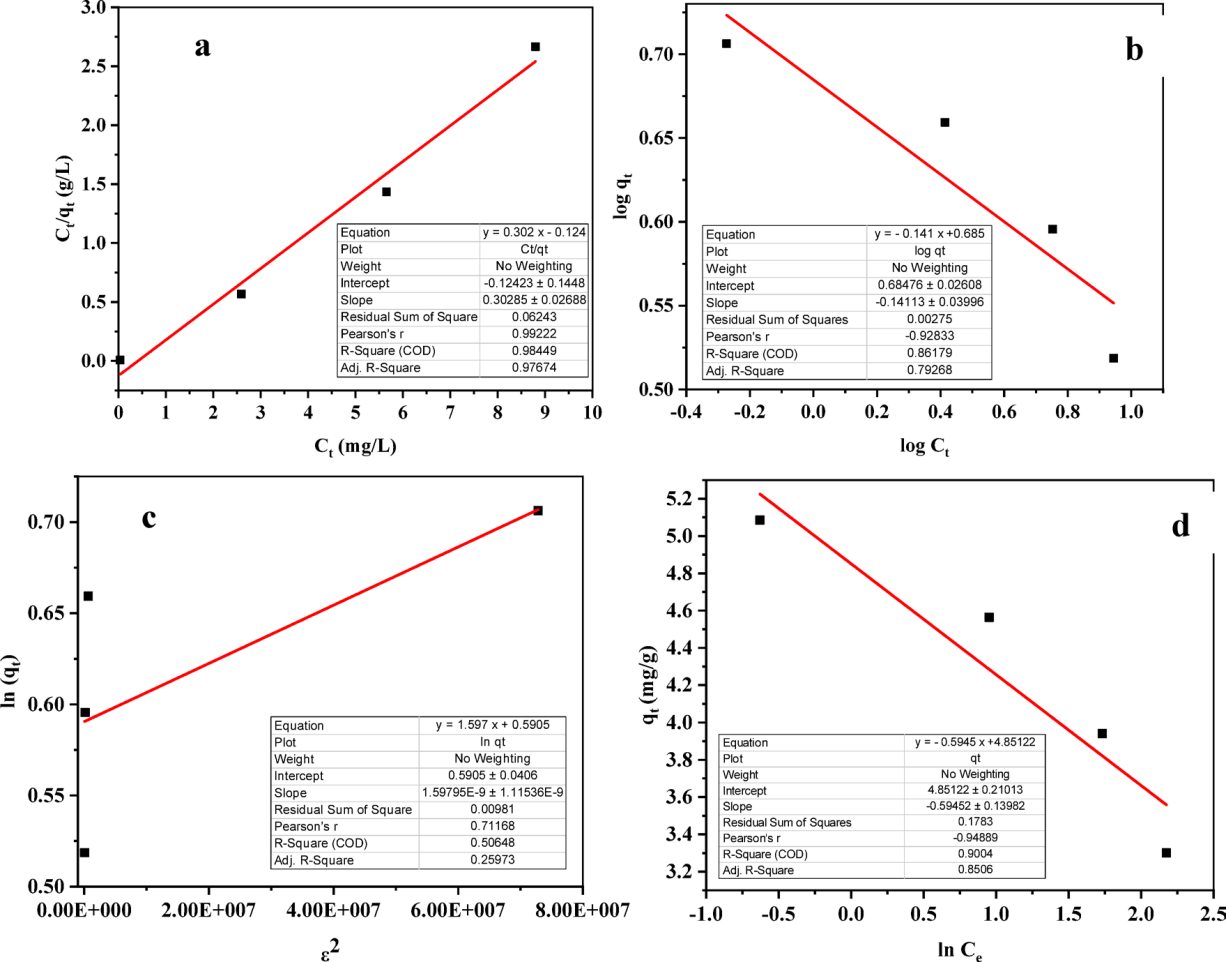
Adsorption isothermal models: (**a**) Langmuir model, (**b**) Freundlich model, (**c**) Dubinin-Radushkevich model, and (**d**) Temkin model.

Among the tested isotherm models (Langmuir, Freundlich, Dubinin-Radushkevich, Temkin), Langmuir provided the best convenient (R²=98.448%) to phenol adsorption on ACES (Fig. 19), implying monolayer coverage on homogeneous sites. This aligns with prior adsorption studies on diverse adsorbents^116,127–131^. Detailed isotherm constants are provided in Table 7.

**Table 7:** Isothermal results of phenol adsorption onto ACES.

Isotherm model	Model parameters	Parameter values	Adj. *R*^2^	*R*^2^ (COD)	RSS
Langmuir model	q_m_, (mg/gm)	3.302	97.674%	98.449%	0.06243
	K_L_, (L/mg)	2.44			
	R_L_ = [1/(1 + K_L_.C_o_)]^132^	0 < 0.0161 < 1 (Favourable)			
Freundlich model	1/n	0.14113	79.268%	86.179%	0.00275
	K_f_, (mg/gm)	4.84			
Dubinin-Radushkevich model	q_max_, (mg/gm)	1.805	25.973%	50.65%	0.00981
	K_DR_, (mole^2^/J^2^)	1.598E-09			
	E, (J/mole)	11180.34			
Temkin model	Ln K_t_, (K_t_ in L/gm)	8.16	85.06%	90.04%	0.1783
	B, (J/mole)	0.5945			

### Thermodynamics of phenol adsorption on ACES

**Fig. 20:**
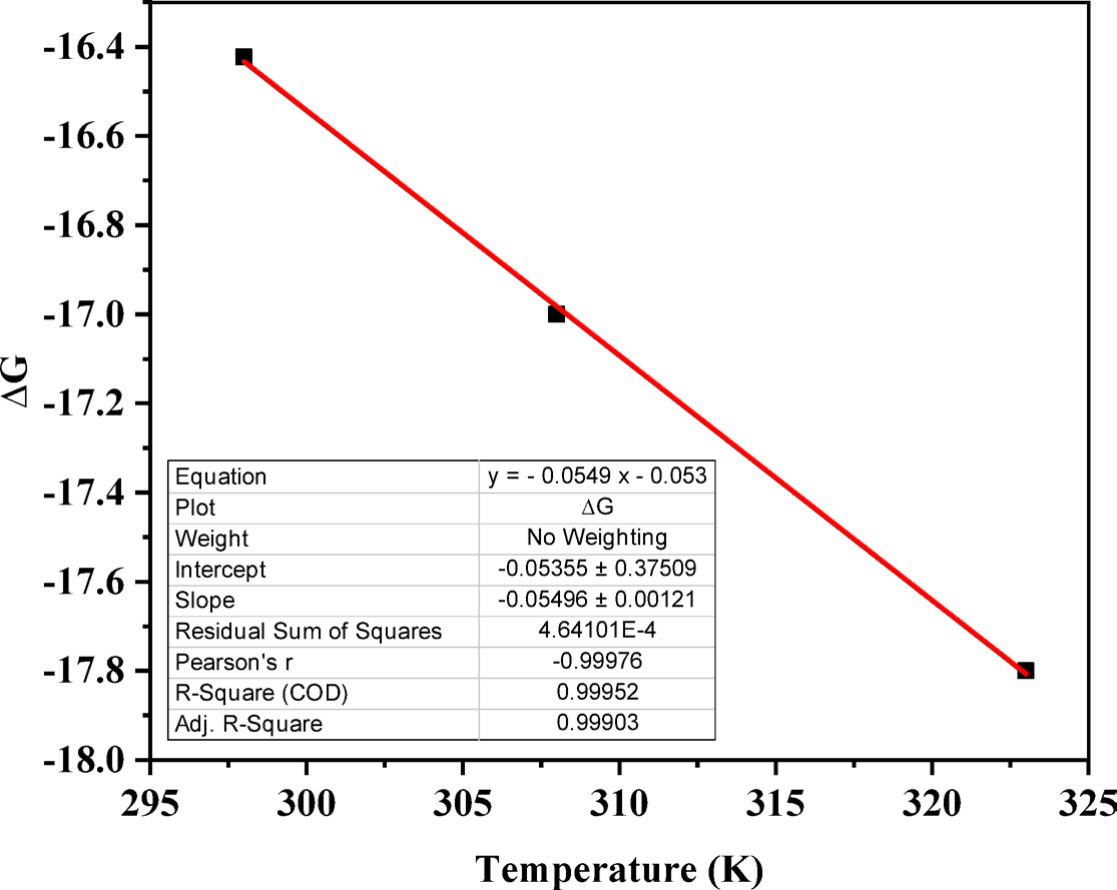
Variation of Gibbs free energy change concerning adsorption temperature.

Thermodynamic analysis of phenol adsorption onto ACES was conducted, focusing on Gibbs free energy change (ΔG), heat of reaction change (ΔH), and entropy change (ΔS). Equations adapted from literature^127,131,133^ were used to calculate these parameters based on equilibrium adsorption capacity (q_e_), equilibrium adsorbate concentration (C_e_), solution volume (V), adsorbent mass (m), gas constant (R), and absolute temperature (T). Figure 20 shows the ΔG vs. T plot, from which ΔH (intercept) and ΔS (slope) were extracted.

ΔG values were negative throughout the studied temperature range, indicating spontaneous adsorption, while an increase in values of ΔG with the rising temperature suggests a decrease in spontaneity of the process^55^. The negative ΔH (0.0535 kJ/mol) revealed an exothermic process, suggesting increased adsorption potential at lower temperatures. This aligns with prior thermodynamic studies on diverse adsorbents^55,56,129,134^.Additionally, the positive ΔS (55.0 J/mol. K) signifies increased randomness at the interface during adsorption, corroborating previous findings^131,135–137^.

#### Regeneration and reusability of ACES adsorbent

**Fig. 21:**
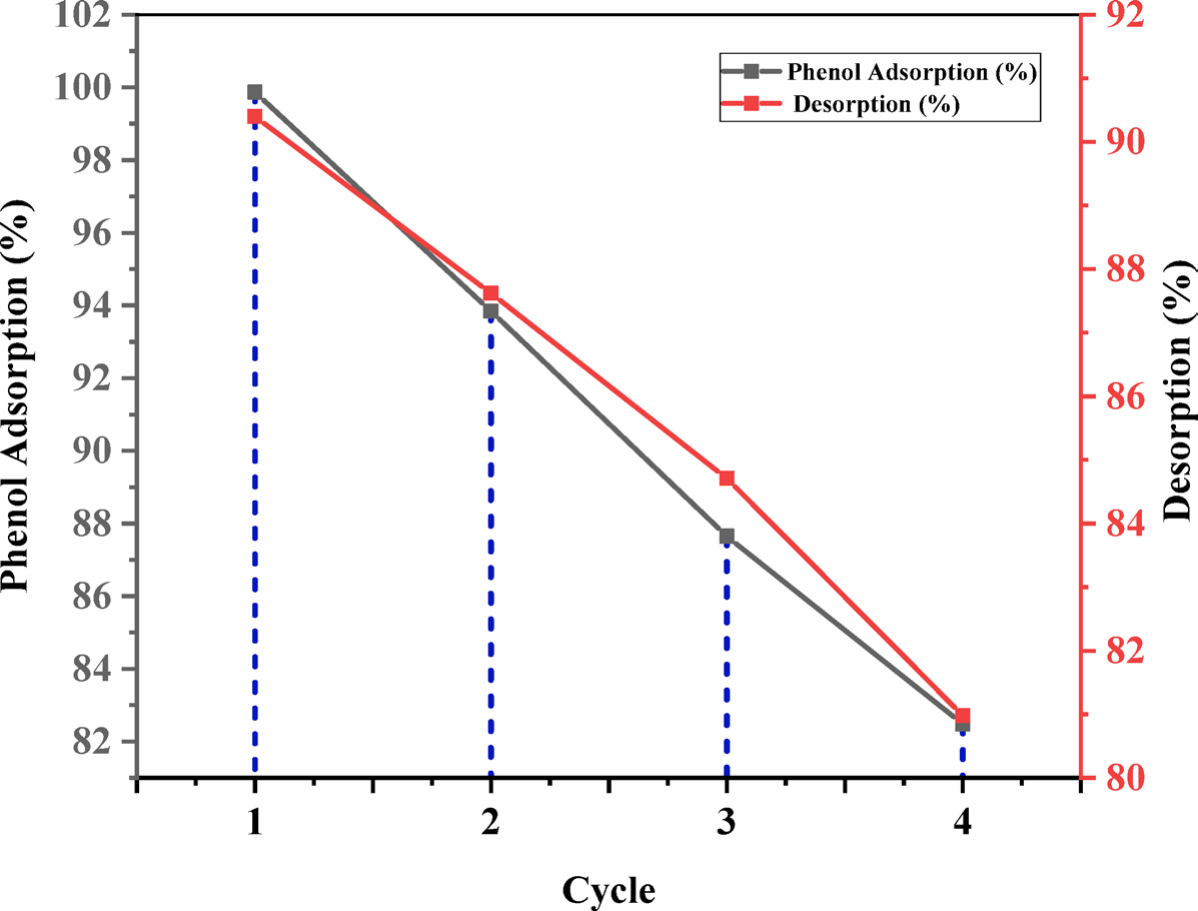
Capability of recycled ACES for phenol adsorption.

Regenerating and reusing activated carbon from eggshells (ACES) is essential for reducing production costs and improving economic viability. The reversibility of the adsorption process and the effectiveness of the adsorption-desorption behaviour of ACES for phenol removal were evaluated using a 0.15 M NaOH solution. The superior regeneration performance of NaOH is attributed to its reaction with phenol, forming a soluble salt, C₆H₅O⁻Na⁺, which aids in desorbing phenol from the adsorbent^127^. After the adsorption process under optimized conditions, phenol-loaded ACES was introduced into 50 mL of 0.15 M NaOH solution. The mixture was agitated at 200 rpm and maintained at 30 °C for the same duration (80 min) as the adsorption experiments until equilibrium was reached. The reaction mixture is then filtered, and the catalyst is washed with distilled water before being dried in the oven to prepare for the next cycle. Following desorption, the concentration of phenol in the solution was determined using a UV–vis spectrophotometer. The percentage of desorption was then calculated using (Eq. 12)^60^. To assess the regeneration potential of ACES, four consecutive adsorption-desorption cycles were conducted using recycled ACES and 0.15 M NaOH as the eluting agent. The removal efficiency slightly drops after four cycles due to a reduction in available adsorption sites. As shown in Fig. 21, ACES maintains excellent performance, with phenol removal efficiency above 80% even after four cycles. However, concerns about its stability over extended cycles, including degradation and blocked adsorption sites, could impact long-term performance. Addressing these issues is essential for evaluating its practical use and identifying areas for improvement in wastewater treatment.

13 $$\text{Desorption percentage }=\frac{\text{Desorped}}{\text{Adsorped}}{*}100$$

Where the desorbed is the concentration of the metal ion in the desorbing solution after the desorption process, and adsorbed is the product of C_o_–C_e_.

#### Differentiation between existing outcomes with earlier consequences

Table 8 summarizes the maximum phenol removal efficiency achieved by different adsorbents. The ACES utilized in this study demonstrates higher adsorption efficiency compared to both natural and modified eggshells reported in the literature. Given its abundance and low cost, ACES offers a significant advantage over other adsorbents.

**Table 8:** A comparison of the phenol removal adsorption efficiency using different adsorbents.

	Adsorbent used	Modification technique	Adsorption parameters optimization methods	A maximum phenol elimination efficiency	Adsorption capacity, mg/gm	References
1	Activated carbons from different components of eggshells (whole shell, shell without membrane, and membrane alone)	Eggshells and their membranes underwent pyrolysis activation at 700 °C for 4 h.	–	–	192 mg/gm	^56^
2	Sawdust-derived activated carbons	Activation of sawdust with eggshells as an activator was carried out at 900ºC.	–	92%	**-**	^138^
3	Calcined Eggshell (CES)	Eggshells were calcined at different temperatures at 200˚C, 400˚C, 600˚C, 800˚C, and 1000˚C for 2 h.	–	37%	–	^57^
4	Eggshell membranes modified biomass to biochar	Eggshell membranes were modified with KOH and HNO_3_ at a carbonization temperature of 300 °C.	–	–	110.38 mg/gm	^139^
5	Wheat straw biochar (BC)	Wheat straw biochar (BC) was acid-washed by HF and activated at 900 ◦C with 10% CO_2_ to obtain biochar.	–	90%	–	^120^
6	ACES	Eggshells are impregnated with H_2_SO_4_ at a 1:2 weight-to-volume ratio and then activated by heating at 650 °C for three hours to complete the activation process.	Design-Expert 13 software and response surface methodology (RSM)	99.87%	5.08 mg/gm	**This study**

## Conclusion

This research highlights the successful production of a high-efficiency activated carbon adsorbent derived from waste eggshells through activation with sulfuric acid. The study employs the Expert design of experiment approach, specifically response surface methodology (RSM), to optimize process parameters for the effective removal of phenol from aqueous solutions using the activated carbon eggshell (ACES). The adsorbent exhibited a BET-specific surface area of 1034.775 m²/g along with considerable porosity, both of which played a key role in its improved adsorption capacity. Phenol removal efficiency of 99.87% was achieved under optimized conditions: initial phenol concentration of 25.015 mg/L, pH 4.693, temperature 25.013 °C, and an adsorbent dosage of 4.913 g.

Key findings include:

1. The adsorption process is predominantly governed by chemisorption, with mechanisms involving electrostatic attraction, π-π interactions, and hydrogen bonding.
2. The Langmuir isotherm model provided the best fit, suggesting monolayer adsorption on homogeneous sites, with the highest convenient (R^2^ = 98.45%).
3. Thermodynamic analysis confirmed the spontaneous and exothermic nature of the process, with increased randomness at the adsorbent-adsorbate interface.

The adsorbent’s performance was comparable to commercial activated carbons, with added advantages of cost-effectiveness and sustainability. Furthermore, ACES retained 80% of its adsorption efficiency after four regeneration cycles, highlighting its practical applicability for wastewater treatment.

This work underscores the potential of utilizing waste materials as sustainable precursors for advanced adsorbents, contributing to environmental management and resource recovery. Future research should focus on scaling up the synthesis process, exploring multi-pollutant adsorption, and conducting long-term reusability studies.

## Data Availability

All data, materials, and software applications or custom code used in this study fully support our published findings and adhere to established field standards. The underlying data supporting the conclusions of this research are accessible upon reasonable request from the corresponding author.
